# Synthesis, antimicrobial activity and conformational analysis of the class IIa bacteriocin pediocin PA-1 and analogs thereof

**DOI:** 10.1038/s41598-018-27225-3

**Published:** 2018-06-13

**Authors:** François Bédard, Riadh Hammami, Séverine Zirah, Sylvie Rebuffat, Ismail Fliss, Eric Biron

**Affiliations:** 1 Faculté de pharmacie, Université Laval and Laboratoire de chimie médicinale, Centre de recherche du CHU de Québec, 2705 Boulevard Laurier, Québec, Québec, G1V 0A6 Canada; 2 STELA Dairy Research Centre, Institute of Nutrition and Functional Foods, Université Laval, Québec, Québec, G1V 0A6 Canada; 3 Molécules de Communication et Adaptation des Microorganismes (MCAM, UMR 7245), Muséum national d’Histoire Naturelle, Sorbonne Universités, CNRS, CP 54, 57 rue Cuvier, 75005 Paris, France; 4 Present Address: School of Nutrition Sciences, University of Ottawa, Ottawa, ON Canada K1N 6N5

## Abstract

The antimicrobial peptide pediocin PA-1 is a class IIa bacteriocin that inhibits several clinically relevant pathogens including *Listeria* spp. Here we report the synthesis and characterization of whole pediocin PA-1 and novel analogs thereof using a combination of solid- and solution-phase strategies to overcome difficulties due to instability and undesired reactions. Pediocin PA-1 thus synthesized was a potent inhibitor of *Listeria monocytogenes* (MIC = 6.8 nM), similar to the bacteriocin produced naturally by *Pediococcus acidilactici*. Of particular interest is that linear analogs lacking both of the disulfide bridges characterizing pediocin PA-1 were as potent. One linear analog was also a strong inhibitor of *Clostridium perfringens*, another important food-borne pathogen. These results are discussed in light of conformational information derived from circular dichroism, solution NMR spectroscopy and structure-activity relationship studies.

## Introduction

Antibiotic therapy was perhaps the most important scientific achievement of the twentieth century in terms of socioeconomic equality and human and animal health. However, misuse of antibiotics led to the emergence of multi-resistant pathogenic strains such as MRSA (methicillin-resistant *Staphylococcus aureus*) and VRE (vancomycin-resistant *Enterococci*) and of health problems due to destruction of commensal microbial flora and even autoimmune diseases^1–5^. There is now an urgent need to develop new agents targeting hitherto unexploited bacterial targets and machineries in order to maintain the ability of modern medicine to treat bacterial infections.

Among the most promising alternatives to conventional antibiotics, bacteriocins have been studied for many years^6–12^. Produced by a wide variety of bacteria to fight other microorganisms in their competitive environments, these ribosomally-synthesized peptides show very promising activities and properties. Hundreds of bacteriocins have been identified and sequenced and are now described in detail in various databases^13,14^. Bacteriocins offer several advantages over other antimicrobial agents used commonly in the food industry and in human and veterinary medicine, including: (i) being safe for consumption, since they are digested completely in the gastrointestinal tract^15,16^; (ii) being 10^3^ to 10^6^ times more potent than several other antimicrobials including conventional antibiotics; (iii) being resistant to common thermal treatments for pasteurization or even sterilization^17–19^. However, despite their great potential and attractive efficacy, the use of bacteriocins remains limited due largely to high production costs (low yield, onerous technological requirements). More research and development and new approaches are needed in order to make the use of bacteriocins as antimicrobial agents feasible on a larger scale, whether in the food industry or in human health and veterinary medicine.

Chemical synthesis has been proposed for the large-scale production of active bacteriocins. However, very few bacteriocins have been successfully prepared in satisfactory yields using such means^20^. Several challenging features that are essential for their bioactivity, such as lasso structure, large macrocycles, presence of lanthionines, glycosylated side chains or complicated peptide motifs all make the task very daunting^21^. Beside their production on a large scale, access to bacteriocins by chemical synthesis would allow further molecular engineering for enhanced potency, improved pharmacological properties, increased stability and modified spectra of activity.

**Figure 1:**
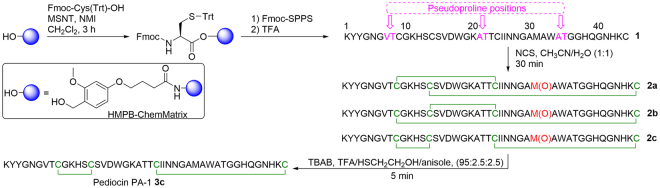
Synthesis of pediocin PA-1 and its analogs.

Among the bacteriocins produced by food-grade microorganisms such as lactic acid bacteria, the class IIa bacteriocins are particularly appealing because they inhibit *Listeria monocytogenes*, a pathogen that causes an illness that is fatal in as many as 20–30% of cases^22^. Genus *Listeria* has been implicated in neonatal deaths, foetal losses, severe meningitis and sepsis^23^. Class IIa bacteriocins contain 35–50 amino acid residues, including one or two cystine bridges (disulfide bonds). The secondary structure of several class IIa bacteriocins, particularly of those containing a single disulfide bond, features an N-terminal β-sheet region followed by a central α-helix and a more disordered C-terminal end^24–28^. Inhibitory activity of class IIa bacteriocins is based on bacterial membrane disruption through specific interaction with membrane-embedded components of the mannose phosphotransferase system^29^. Among members of the class IIa, we were particularly interested in pediocin PA-1, a peptide of 44 amino acid residues containing two disulfide bonds between Cys9–Cys14 and Cys24–Cys44 (Fig. 1, **3c**)^30^ and proposed to be ensured by an accessory protein that seems to display a chaperone-like activity in conjunction with an ABC transporter during secretion^31^. The Cys9–Cys14 bond is conserved among the class IIa bacteriocins, as is the N-terminal YGNGV sequence, both structures being signatures of this family^26^. Despite recent progress in its production by recombinant technologies^32^, pure bioactive pediocin PA-1 is not commercially available at present time and commercial crude fermentation products such as ALTA 2341^®^ (Quest International)^9^ or freeze-dried pediocin-producing cultures like CHOOZIT^™^ FLAV 43 (Danisco) must be used^33^. Total chemical synthesis of bioactive pediocin PA-1 has been attempted in the past but with limited success, due to yields below 1% after multiple purification steps^20,34^. More recently, designed analogs of pediocin PA-1 have been synthesized in low yields (0.3–3.8%), but these have shown reduced or no activity compared to the native bacteriocin^34–36^. Despite the great potential of pediocin PA-1 as an antimicrobial agent, the problems associated with its production continue to limit its applicability and delay regulatory approval.

To pave the way for the future use of pediocin PA-1 as a food preservative and substitute for antibiotics, we report here the synthesis of bioactive pediocin PA-1 and several analogs thereof using a combination of solid- and solution-phase synthesis strategies. We show that pediocin PA-1 and two linear analogs lacking disulfide bonds or other rigidifying motifs have a similar activity against *Listeria* spp. target organisms^35^. Using a panel of bacterial strains, we show in addition that one linear analog is also a strong inhibitor of *Clostridium* spp. We also compare the conformational features of class IIa bacteriocins stabilized by two disulfide bonds to those containing none.

## Results

### Total synthesis of pediocin PA-1 and analogs

The peptides were prepared by standard solid-phase peptide synthesis (SPPS) using the Fmoc/tBu strategy (Table 1)^37,38^. The HMPB-ChemMatrix resin was selected because of its higher performance with larger peptides and ability to form aggregation-disrupting interactions with growing peptide chains^39^. A first attempt to prepare the linear precursor **1** by stepwise amino acid additions yielded a complex mixture of short peptides but not the desired product. To identify the problematic amino acid couplings, a series of C-terminal ladder sequences starting from Gly40 (GNHKC, QGNHKC, etc.) were prepared in parallel and analyzed by LC-MS after cleavage from the resin. The results showed that coupling Fmoc-Asn(Trt)-OH to Gly29 was ineffective. To overcome difficult couplings and prevent aggregation during peptide elongation, the pseudoproline strategy was used^38,40^. As proposed previously by Vederas and coll. for leucocin A and pediocin PA-1 analogs^35^, pseudoprolines were incorporated at the critical positions by coupling Fmoc-Val-Thr(Ψ^Me,Me^ pro)-OH with residue Cys9 and Fmoc-Ala-Thr(Ψ^Me,Me^ pro)-OH with residues Thr23 and Gly36 (Fig. 1). Finally, the combination of the HMPB linker, ChemMatrix resin and pseudoprolines in SPPS allowed isolation of linear pediocin PA-1 **1** with 70–80% crude purity after side chain deprotection and cleavage from the resin. However, in addition to the desired compound, the crude product also contained side chain-alkylated peptides as impurities, based on LC-MS analysis. Their formation was prevented using treatment with suitable TFA cocktails to obtain the linear peptide **1** as a single peak in 45% yield after HPLC purification (Fig. S1).

**Table 1:**
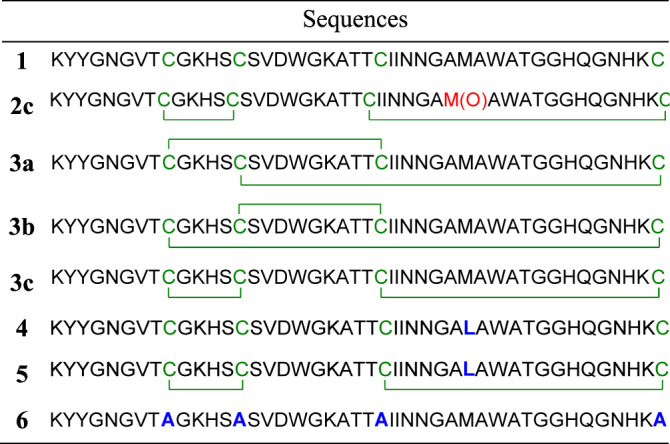
Peptides synthesized for antibacterial assays and conformational studies.

**Figure 2:**
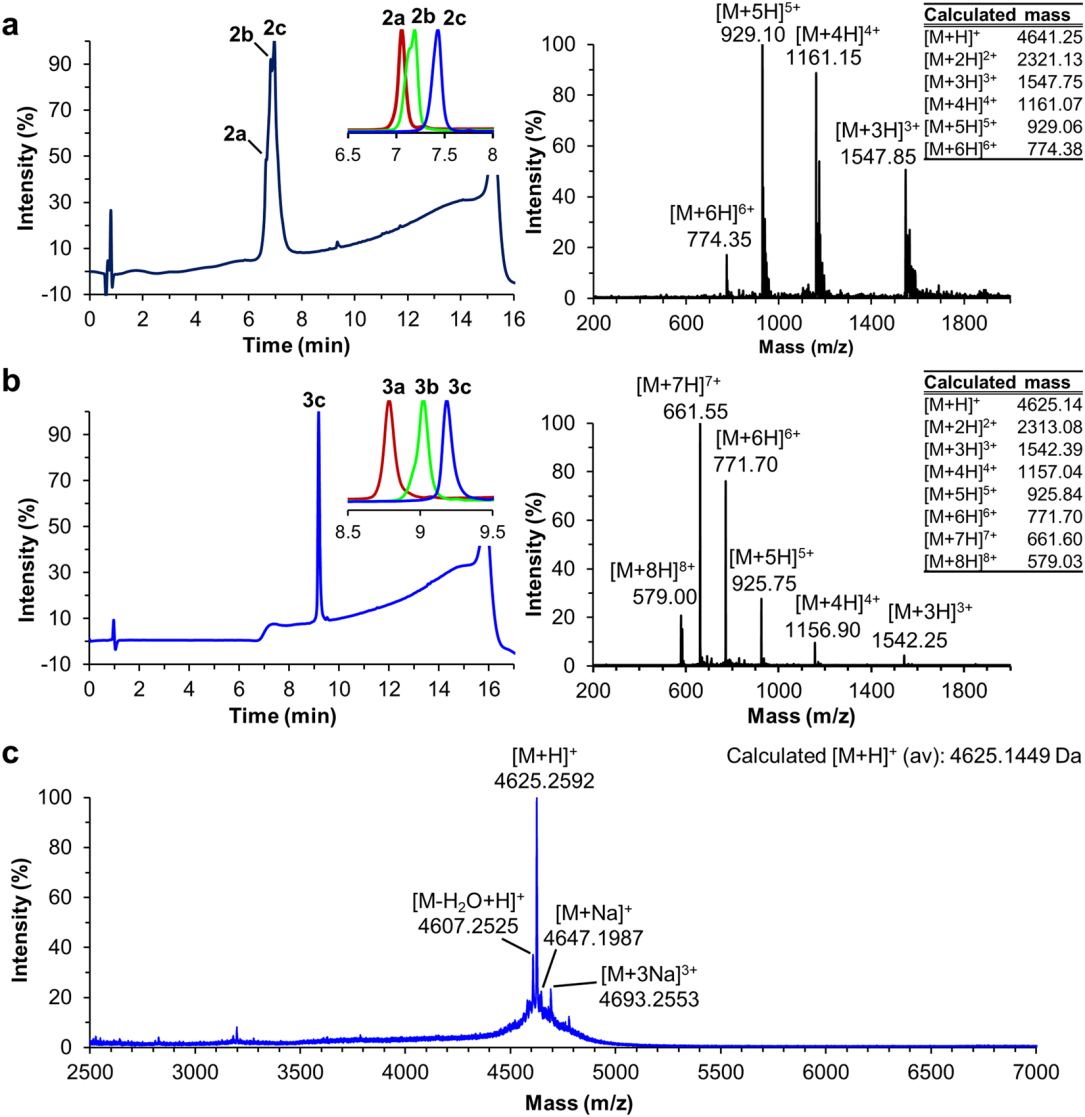
HPLC and ESI-MS profiles of (**a**) crude oxidized pediocin PA-1 **2a–c** (overlaid HPLC traces of purified compounds are shown in the inset) and (**b**) purified pediocin PA-1 **3c** (overlaid HPLC traces of purified **3a**, **3b** and **3c** are shown in the inset). (**c**) MALDI-TOF MS spectrum of synthetic pediocin PA-1 **3c**.

Correct pairing of the thiol groups to form the disulfide bonds found in natural pediocin^30,31^ was achieved under mild oxidative conditions to generate the desired compound **3c**. Since the Met31 residue is very sensitive to aerobic oxidation and pediocin PA-1 oxidized at this position (**2c**) is 100 times less active^41^, different methods for disulfide bond formation were evaluated. Unfortunately, oxidation of Met31 was not prevented under any of the conditions tested^42,43^. We therefore opted to perform disulfide bond formation and methionine oxidation simultaneously with *N*-chlorosuccinimide (NCS)^44^, followed by selective reduction of the Met31 side chain (Fig. 1). In initial attempts to obtain peptides **2a–c** with the different possible disulfide bridge arrangements from linear precursor **1** using the procedure with NCS^44^, many side reaction products were observed in the HPLC chromatogram. Analysis of MS and MS/MS data showed that most of these were peptides containing chlorinated aromatic side chains. It has been reported that TFA anions can catalyze the halogenation of aromatic residues by *N*-halogeno-succinimides^45^. To avoid the occurrence of a similar reaction in the presence of NCS, acetic acid was used in the mobile phase instead of TFA during HPLC purification of linear precursor **1**. Under these conditions, chlorine adducts were not observed and the oxidized Met31 bicyclic analog **2** was obtained in three different disulfide bond combinations (Fig. 2a). After purification by HPLC (Fig. S2), the peptides were obtained in the following proportions: **2a** 7.5%, **2b** 21.5% and **2c** 71.0% and the third peak was assumed to be the oxidized native pediocin PA-1 **2c**, as reported by Oppegård *et al*.^31^. The last challenge of our approach was to selectively reduce the Met31 oxidized side chain without affecting the disulfide bonds. This was achieved using anisole and tetrabutylammonium bromide (TBAB) in TFA at room temperature as described by Taboada *et al*.^46^ but with the addition of β-mercaptoethanol (Figs 1 and S3). Isolation of pediocin PA-1 (**3c**) was confirmed by LC-MS (Fig. 2b) and MALDI-TOF MS with the expected molecular ion being observed at m/z 4625.2592 (calculated [M + H]^+^ (av) for C_196_H_294_N_61_O_60_S_5_: 4625.1449Da) (Fig. 2c). The amino acid sequence of synthetic pediocin PA-1 **3c** was then validated by MS/MS after disulfide bond reduction, cysteine *S*-alkylation with iodoacetamide and trypsin digestion (Fig. S4) and the correct disulfide bond pairing of **3c** confirmed by LC-MS/MS analysis of peptides **3a**–**c** (Fig. S5). By combining different solid- and solution-phase methodologies, we were able to obtain pediocin PA-1 **3c** in 11% overall yield. The same procedure was applied to purified peptides **2a** and **2b** to afford peptides **3a** and **3b**, respectively. In order to study the bioactivity without interference due to oxidation of Met31 during synthesis, antibacterial assays and conformational studies^35,36^, a linear analog of pediocin PA-1 containing a Leu31 residue (substitution of Met31 with Leu) was prepared according to the procedure described above. After purification, compound **4** was obtained in 55% yield and subjected to disulfide bond formation with NCS to afford compound **5**, the pediocin PA-1 analog M31L (Table 1 and Fig. S6). The reduction protocol was skipped in this case. Finally, in order to determine whether or not disulfide bonds are essential for antimicrobial activity and for maintaining the bacteriocin in its bioactive conformation, the cysteine residues were replaced by alanine, giving analog **6**.

### Antimicrobial activity of pediocin PA-1 and its analogs

**Figure 3:**
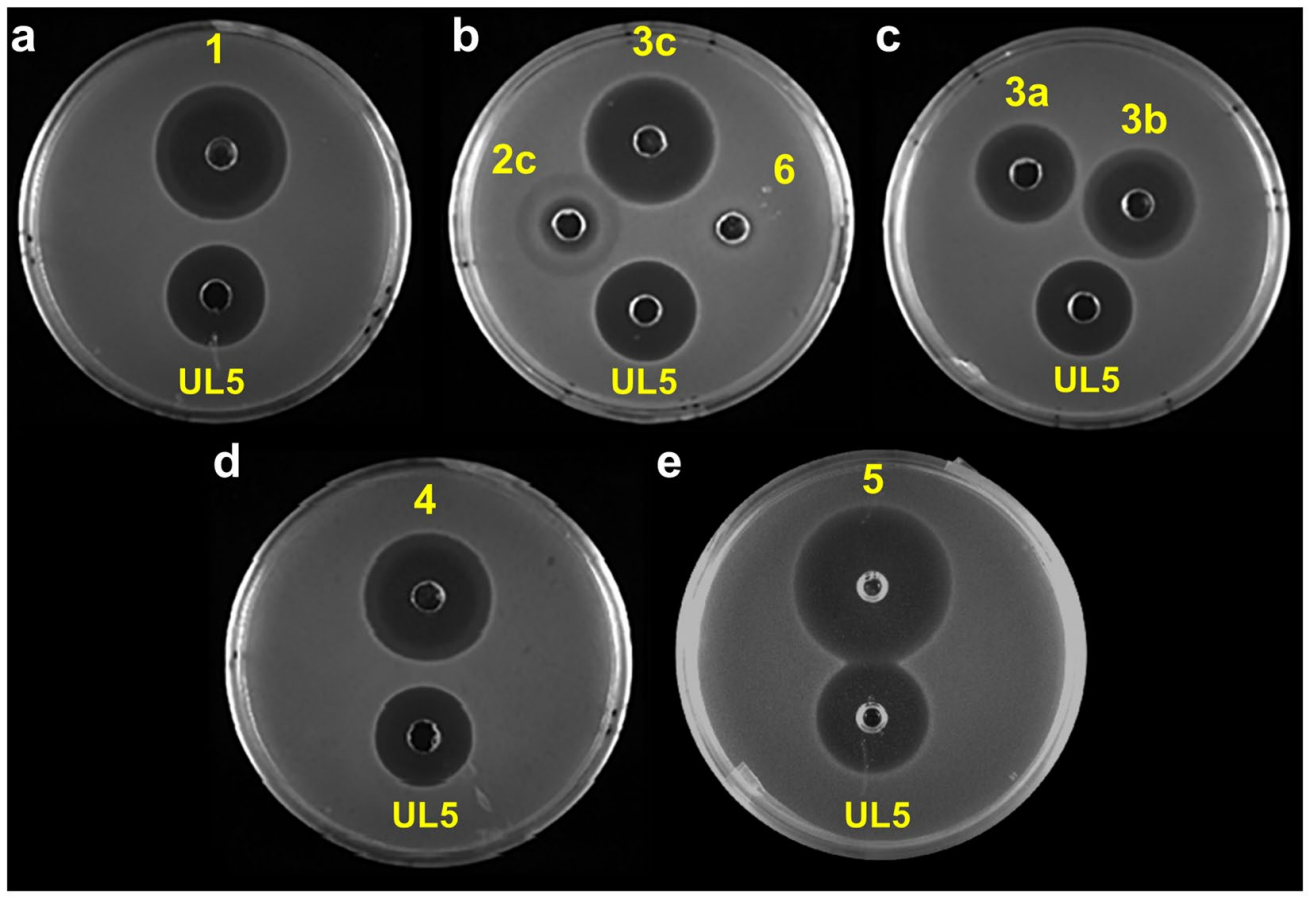
Antimicrobial activity of natural pediocin PA-1 produced by *P. acidilactici* UL5 and synthesized peptides **1**, **2c**, **3a**–**c**, **4** and **5** against *L. monocytogenes* ATCC 19111 in soft agar TSBYE medium (**a**–**e**). Inhibition zone diameters (mm) were respectively 32, 14, 24, 27, 31, 31 and 34. Volume of UL5 culture supernatant or solution of synthetic compound (1 mg/mL) placed in agar wells was 75 µL.

The antibacterial activity of the synthesized peptides was first assessed using radial diffusion assays with *L. monocytogenes* ATCC19111 relative to native pediocin PA-1 produced by *Pediococcus acidilactici* UL5 as described previously (Fig. 3)^16^. The inhibition zone diameter obtained using *P. acidilactici* UL5 culture supernatant was 24 mm (Fig. 3a–e). As expected, inhibition by synthetic pediocin PA-1 **3c** was substantial (31 mm), while little and no activity were observed for analogs **2c** and **6** respectively (Fig. 3b). Compared to synthetic pediocin PA-1 **3c**, the activity was reduced for analogs **3a** and **3b**, with inhibition diameters of 24 and 27 mm respectively (Fig. 3c). To our surprise, linear analogs **1** and **4**, lacking disulfide bonds in the solid state prior to the bioassay, showed excellent activity with inhibition diameters of 32 mm, similar to pediocin PA-1 **3c** (Fig. 3a,d). Finally, the M31L analog **5** was also a strong inhibitor of *L. monocytogenes* LSD530, with an inhibition diameter of 34 mm, similar to those of **1**, **3c** and **4** (Fig. 3e).

The antimicrobial activity of peptides **1**, **2c**, **3a**–**c**, **4**, **5** and **6** was then assessed in terms of minimal inhibitory concentrations (MIC) against *L. monocytogenes* LSD530, *L. monocytogenes* ATCC 19111, *L. ivanovii* HPB28 and *P. acidilactici* UL5 (Table 2). Synthetic pediocin PA-1 **3c** showed strong inhibition, with low nanomolar MICs of 6.8 nM against *L. ivanovii* HPB28 and *L. monocytogenes* LSD530 and 13.5 nM against *L. monocytogenes* ATCC 19111. Compared to pediocin PA-1 **3c**, peptides **3a** and **3b**, which have incorrect disulfide bond pairings, were 2–4 times less active (MIC 13.5–27 nM). The observed activity could be explained by an equilibrium in disulfide bond pairing that ultimately would lead to the most stable and active conformation of pediocin PA-1 **3c** for a portion of the peptide. As expected, a significant decrease of activity was observed for analog **2c** (oxidized Met31), with MICs between 1562 and 25000 nM. These results confirmed that oxidation of Met31 plays a crucial role in the potency of pediocin PA-1^41^. Meanwhile, linear analogs **1** and **4** showed an activity comparable to that of **3c**, with MICs ranging from 6.8 to 13.5 nM against the same target strains, in agreement with the agar diffusion results. In addition, the same range of MICs was observed for the M31L analog **5**. Overall, the antimicrobial activity observed here for synthetic pediocin PA-1 **3c** and for its active analogs **1**, **4** and **5** is in agreement with the MICs reported previously for the bacteriocin obtained by fermentation^41^. Furthermore, the linear analog **6** was completely inactive, demonstrating that disulfide bonds are indeed essential for pediocin PA-1 activity. Finally, pediocin PA-1 **3c** and the synthesized analogs were inactive against *P. acidilactici* UL5, a pediocin producer immune to the bacteriocin.

**Table 2:** Minimal inhibitory concentrations of synthetic pediocin PA-1 **3c** and its analogs on *Listeria* strains and the producer *P. acidilactici*.

Strain	Minimal inhibitory concentration (nM)							
	**1**	**2c**	**3a**	**3b**	**3c**	**4**	**5**	**6**
*L. ivanovii* HPB28	6.8	1562	27.0	13.5	6.8	6.8	1.7	n.a.^a^
*L. monocytogenes* LSD530	13.5	1562	27.0	13.5	6.8	13.5	6.8	n.a.
*L. monocytogenes* ATCC 19111	13.5	25000	27.0	13.5	13.5	13.5	13.5	n.a.
*P. acidilactici* UL5	n.a	n.a	n.a	n.a	n.a	n.a	n.a.	n.a.

^a^n.a. = no activity detected at concentrations up to 100 µM.

### Spectrum of activity of pediocin PA-1 analog 4

Based on the mode of action of pediocin PA-1 and its ability to bind the mannose phosphotransferase IICD complex (manPTS IICD)^47,48^, a protein Blast with the sequence of manPTS IID from *L. monocytogenes* (WP_003721724.1) was performed to identify other potential sensitive strains. The study showed that manPTS IID is a highly conserved sequence among a wide variety of bacteria, and we were able to sort 175 species from the generated phylogenetic tree (Fig. S7). From these results, 12 strains found in the tree (*Bacillus cereus*, *Bacillus coagulans*, *Carnobacterium divergens*, *Clostridium perfringens* (2), *Enterococcus faecalis*, *Enterococcus hirae*, *Lactobacillus plantarum*, *Lactobacillus salivarius*, *Leuconostoc mesenteroides, Listeria murrayi*, *Listeria seeligeri*), 5 strains similar to those in the tree (*Bacillus subtilis*, *Clostridium tyrobutyricum*, *Lactobacillus acidophilus*, *Lactococcus lactis*, *Streptococcus thermophilus*) and 5 strains outside the tree (*Bifidobacterium animalis*, *Escherichia coli*, *Staphylococcus aureus* (3)) were selected and used in antimicrobial assays with the linear M31L analog **4** (Tables 3 and S1). Since analog **4** resists oxidation better and showed equivalent activity to the native peptide in antimicrobial assays, it was used instead of **3c** to explore the spectrum of activity. Of the 22 tested strains, 7 strains all found in the phylogenetic tree were sensitive to analog **4** (Table 3 and Fig. S8). The strongest activity was obtained against *C. divergens* and *L. seeligeri*, with MIC at 1.9 and 4.7 nM, respectively. Peptide **4** also showed a strong activity against *C. perfringens*, with MICs ranging from 37.8 nM to 75.7 nM. No strains lying outside the phylogenetic tree or with a high degree of similarity showed sensitivity to the synthetic analog **4** (Table S1). Antimicrobial activity of pediocin PA-1 against *Micrococcus luteus* has been reported recently^49^. However, we found no activity against *M. luteus* ATCC 10240 for synthetic pediocin PA-1 **3c** or its analogs at up to 100 µM.

**Table 3:** Spectrum of activity of synthetic analog **4**: minimal inhibitory concentration against selected sensitive strains.

Strain	Reference	MIC^a^ (nM)	Diffusion assay^b^ (mm)	Culture medium
*Carnobacterium divergens*	ATCC 35677	1.9	35	BHI
*Leuconostoc mesenteroides*	ATCC 23386	1.9	33	MRS
*Listeria seeligeri*	ATCC 35967	4.7	32	TSBY
*Clostridium perfringens*	AAC 1–222	37.8	25	RCM
*Clostridium perfringens*	AAC 1–223	75.7	22	RCM
*Listeria murrayi*	ATCC 25401	151.4	28	TSBY
*Lactobacillus plantarum*	ATCC 8014	605.5	23	MRS

^a^Determined from micro-dilution assay.

^b^Inhibition zone diameter.

### Conformational studies by circular dichroism

**Figure 4:**
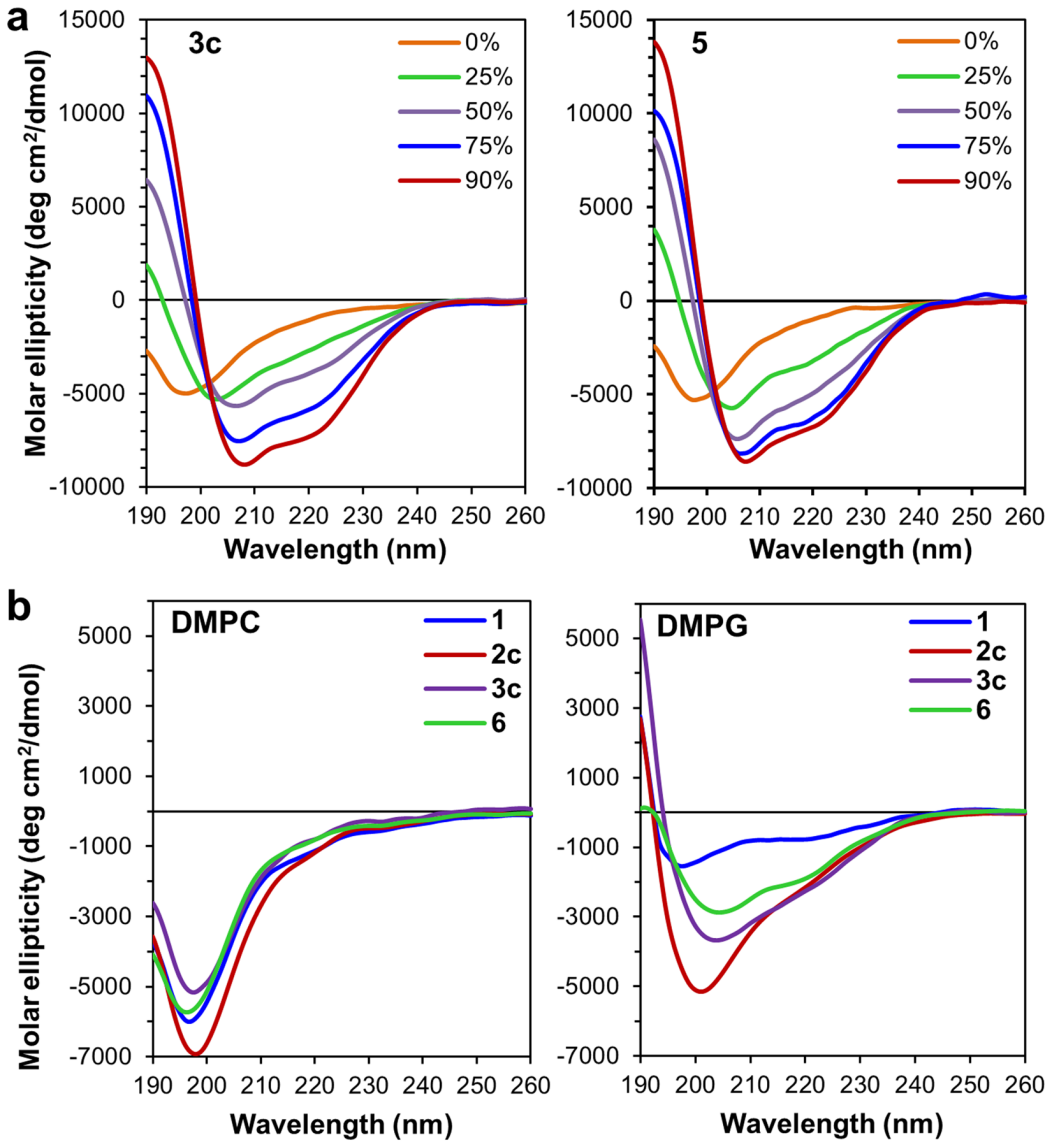
Circular dichroism spectra of synthetic pediocin PA-1 **3c** and its analogs **1**, **2c** and **6**. (**a**) **3c** (left) and **5** (right) in various percentages of TFE and (**b**) **1**, **2c**, **3c**, and **6** in DMPC (left) and DMPG (right) vesicles with a peptide/lipid ratio of 1/100.

Circular dichroism (CD) in the far-UV region (260–190 nm) provides information on the secondary structure (α-helix, β-sheet) of a protein or a peptide in a particular medium^50^. Since class IIa bacteriocins are known to be unstructured in aqueous solution and to acquire their three-dimensional structure in the presence of membrane-like environments, CD was used to compare the different conformational features of synthetic pediocin PA-1 **3c** and its analogs in different media (Figs 4 and S9). It also allowed us to determine the best conditions for further conformational NMR studies. Several class IIa bacteriocins have been analyzed previously in H_2_O/TFE solvent systems at 50–90% TFE, which favour peptide structuration^25,28,51^. It has also been reported that TFE induces β-sheet structure in the N-terminal region of class IIa bacteriocins^26^. Analysis of our CD data revealed that the pediocin PA-1 analogs are unstructured in water and in DMPC vesicles, but tend to become structured in the presence of DMPG, as observed previously^52^ and in TFE at 298 K (Fig. 4 and Table S2). Structuration of the peptides appeared stronger in the presence of TFE than in DMPG vesicles. In 50% TFE, the secondary structure of pediocin PA-1 **3c** was well balanced between α-helix (22.2%), β-strand (23.9%), turn (21.8%) and coil (32.1%), compared to the results obtained in other mixtures (Table S2) and also in the range of 20–30% α-helix expected for class IIa bacteriocins. This environment was therefore selected for the NMR study.

### NMR spectroscopy and structure analysis

Low pH values (<3, e.g. ~2.8 in 0.1% TFA) are required in order to maintain peptide disulfide bridges in right pairs, since oxidation occurs in the pH 3–8 range^53^. The M31L analog **5** was therefore selected for NMR measurements in order to avoid Met31 oxidation and observe the structures most representative of active pediocin PA-1 during data acquisition. Leucine is considered to be a suitable replacement for methionine since it provides a hydrophobic residue with similar properties and structure^54^. A series of one-dimensional ^1^H NMR spectra at different temperatures allowed selection of the optimal temperature of 313 K (Fig. S10) both to prevent disulfide bond breakage and to obtain enhanced resolution for further two-dimensional homonuclear ^1^H-^1^H total correlation spectroscopy (TOCSY) and nuclear Overhauser effect spectroscopy (NOESY) experiments (Fig. S11). The CS-Rosetta software was used for this study because it has been reported to determine very accurately the structure of proteins up to 40 kDa with only backbone chemical shifts^55,56^ as well as that of smaller proteins with very high sequence identity but completely different structures with only ^1^H^N^ and ^1^H^α^ chemical shifts^57^. The quality of the spectra allowed 87% of the ^1^H signals (219 of 250) to be assigned manually (Table S3). ^1^H chemicals shifts, sequence and disulfide bridge constraints were used as input into the software to determine the final NMR structure using 40,000 different conformers. Convergence of the conformers to the lowest energy structure was observed (Fig. S12). To verify that the correct disulfide bridge pairings imposed as constraints during calculation did not introduce a bias to the final structure, calculations were performed in parallel with the two other pairings (C9–C24/C14–C44 and C9–C44/C14–C24). By contrast, these calculations led to structures without any convergence (Fig. S13). Viability of the structure generated from the first calculation was confirmed from different NOE assignments (Table S4). Finally, the localization of the different types of structures in the CS-Rosetta generated global structure was compared with that deduced from the chemical shift deviation (CSD) calculated for pediocin PA-1 M31L from the random coil values of amino acid chemical shifts^58^. The method proved powerful previously for deciphering and assigning different structure types to regions in peptides and proteins and was applied to other type IIa bacteriocins^24,26^. The calculated CSD (Fig. S14) appeared to nicely fit with those of type IIa bacteriocins devoid of the second disulfide bond^24,26^ and were in very good agreement with the structure predicted in the present study.

**Figure 5:**
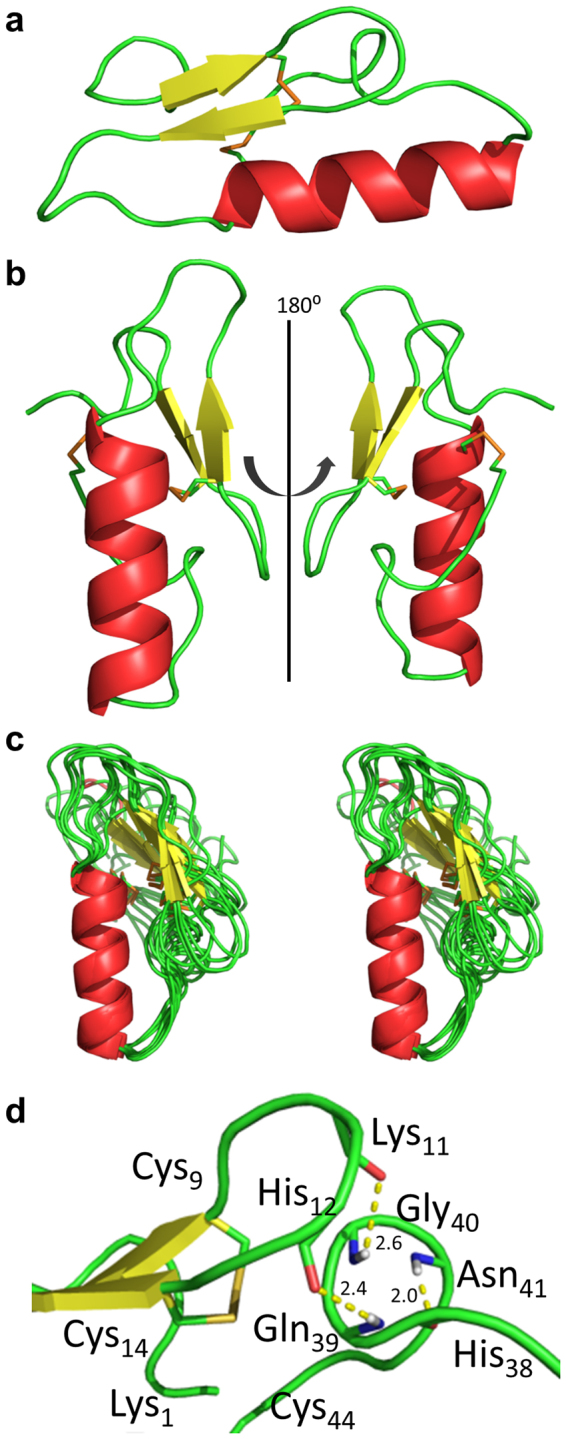
(**a**) and (**b**) NMR lowest relative energy structure (α-helix red, β-sheet yellow, loops green and disulfide bridge orange) of synthetic pediocin PA-1 M31L analog **5** obtained from chemical shifts (H_2_O/TFE-d2 (1:1, v/v) at 313 K) and sequence with CS-Rosetta. (**c**) 10 lowest energy structures aligned with the helix (T23 to T35) in cross-eyed view. (**d**) Close-up of the interaction between the N-terminal β-turn and the C-terminal 3_10_ helix. CO_His38_-NH_Asn41_ 2.0 Å; CO_His12_-NH_Gln39_ 2.4 Å; CO_Lys11_-NH_Gly40_ 2.6 Å. PDB entry: 5UKZ.

The generated structure of the M31L analog **5** (Fig. 5a,b) is very similar to structures determined previously for other class IIa bacteriocins. It consists of an N-terminal antiparallel β-sheet stabilized by the Cys9–Cys14 disulfide bond and a C-terminal tail folded into an α-helix, which folds back onto the β-sheet to form a hairpin-like structure. The tryptophan residues at positions 18 and 33 are located on both sides of the helix. These two distinct regions are connected to the conserved flexible hinge formed with Asp17^26^. CD results showed a α-helix of similar size with 13 residues (29.5%). Figure 5c shows a stereo view of the superimposed structure for the 10 lowest energy structures, of which the quality was evaluated (Fig. S15)^59^. The definition of the aligned α-helix has a RMSD of 0.26 Å between residues 23 to 35 of the α-helix (the 13 backbone Cα) and a total RMSD of 1.89 Å for the 1–44 Cα (Fig. S16). Calculated RMSD of the C-terminal tail from residues 36 to 44 shows an average of 1.57 RMSD and tends to conserve a single 3_10_ helix loop where the N–H of the Asn41 forms a hydrogen bond with the C=O of His38 with a distance of 2.0 Å. While no well-defined structure is associated with the N-terminal β-sheet, presumably due to a lack of information in this region, the presence of two strong hydrogen bonds between the β-hairpin loop and the 3_10_ helix (His12-Gln39; 2.4 Å, Lys11-Gly40; 2.6 Å) suggests that it interacts with the C-terminal chain to fold the peptide into a more rigid conformation (Fig. 5d). It has been reported that the second disulfide bridge allows greater potency at higher temperatures^60^, and a strong interaction between the N and C terminals could explain this.

Class IIa bacteriocins have been subdivided into three groups determined from sequence alignment^61^. So far, 3D structures have been determined for only 7 class IIa bacteriocins: sakacin P, sakacin P N24C + 44C^26^, enterocin HF^28^, pediocin PA-1 M31L (group 1), leucocin A (group 2)^24^, carnobacteriocin B2^25^ and curvacin A (group 3)^27^. Each subgroup has at least one determined structure. It is interesting that only groups 1 and 2 have the N-terminal antiparallel β-sheet, which contains the conserved YGNGV sequence and the N-terminal disulfide bond.

## Discussion

Our goal was to develop a convenient route for pediocin PA-1 synthesis and give access to analogs with possible improved stability, increased potency and modified or extended spectrum of activity. The chemical synthesis of this bacteriocin is known to be difficult and fraught with pitfalls. The present study showed that the combined use of three pseudoproline dipeptides distributed throughout the amino acid sequence and PEG-based resin as solid support was important for successful preparation of linear pediocin PA-1 by standard solid-phase peptide synthesis. We found that adding phenol to the TFA cocktail eliminated side chain alkylation reactions observed during cleavage from the resin and the deprotection step. Replacing TFA with acetic acid in the mobile phase during HPLC purification prevented chlorine adduct formation during disulfide bond formation with NCS. Finally, after disulfide bond formation, the oxidized Met31 could be reduced selectively using β-mercaptoethanol in TFA with anisole and tetrabutylammonium bromide. The combination of these adjustments allowed us to avoid several pitfalls and obtain pediocin PA-1 (**3c**) with an overall yield of 11%. These results represent a significant improvement compared to a previously reported synthesis where a cation-exchange chromatography and three HPLC purification runs were needed to obtain the peptide in yields of less than 1%^34^. The described approach was then applied to prepare the pediocin PA-1 analogs examined in this study.

As expected, synthetic pediocin PA-1 **3c** strongly inhibited *L. ivanovii* and *L. monocytogenes*, giving large inhibition zone diameters in the radial diffusion assay (Fig. 3b) and low nanomolar MIC in the micro-plate assay (Table 2). While the oxidized Met31 analog **2c** (Fig. 3b) was inactive, the M31L analog **5** was nearly as active as **3c** (Table 2 and Fig. 3e). These results suggest that the role of Met31 in the molecular recognition and mechanism of action of pediocin PA-1 is primarily the provision of a hydrophobic binding site. In contrast, incorrect disulfide bond pairings such as in analogs **3a** and **3b** (Fig. 3c) reduced inhibitory activity. These results suggest that disulfide bonds may exist in a dynamic equilibrium that allows some remodelling in the culture medium to produce a small quantity of the bioactive conformation. Disulfide bridge formation is well known to occur as a dynamic reversible process with any thio/disulfide pair acting as a redox buffer^62^. What is surprising in the present study is that equivalent antimicrobial activities were observed for linear analogs **1** and **4** in radial diffusion (Fig. 3a and d) and micro-plate dilution assays (Table 2). These results support the *in situ* disulfide bond formation hypothesis proposing that suitable disulfide bonds can be formed in the bioassay medium without the help of chaperone-like proteins^31^. Based on the disulfide bonds pairing ratios of **2a** (7.5%), **2b** (21.5%) and **2c** (71.0%) obtained after cyclization with NCS and the similar antimicrobial activities, the same equilibrium could be reached in culture medium containing linear analogs **1** and **4** but also native pediocin PA-1 **3c**. Substitution of all four cysteine residues with alanine showed that the disulfide bonds are essential for pediocin PA-1 activity, since linear analog **6** was inactive (Table 2 and Fig. 3b). As previously reported with norleucine mutants^35,36^, the substitution of Met31 by a leucine residue in analogs **4** and **5** did not reduce the antimicrobial activity but greatly improved the molecular stability and, in our case, significantly increased overall isolated yields. Finally, neither pediocin PA-1 **3c** nor any of its analogs were active against the pediocin producer *P. acidilactici* UL5, as expected.

With anti-listeria activity equivalent to pediocin PA-1 **3c**, the linear M31L analog **4** is the most appealing compound for future applications in the food industry, veterinary medicine and the biomedical sector. Without the need for disulfide bond formation prior to use, this peptide could be produced in larger quantities and significantly higher yields since one synthetic step (oxidative cyclization) and one purification step are avoided in the process. Compared to the 11% yield observed for pediocin PA-1 **3c**, analog **4** was obtained with an overall yield of 55% after purification. Moreover, in addition to the superior yield and lack of the sensitive Met31, the stability of analog **4** in solution during storage, handling and assays was significantly greater. For these reasons, analog **4** was used in assays against the strains selected from the *L. monocytogenes* manPTS IID protein Blast. This screening allowed identification of seven sensitive strains, all found in the phylogenetic tree (Table 3 and Fig. S7). In addition to the expected anti-listerial activity, the most interesting result from this focused screening was certainly the strong activity of analog **4** against the pathogen *C. perfringens* with MICs of 37.8–75.7 nM in micro-plate assays and inhibition diameters of 22–25 mm in radial diffusion assays (Table 2 and Fig. S8). Previously reported inhibitory effect of pediocin PA-1 and pediocin-producing *P. acidilactici* against *C. perfringens* in frankfurters and dry-fermented sausages support our results^63^. Since *C. perfringens* is one of the most common causes of food poisoning in the United States (about 250,000 cases of food borne illness every year in North America)^64,65^, the observed strong potency of analog **4** against this bacterial strain makes it very attractive for future applications. Furthermore, a total of 175 strains were sorted from the protein Blast and further antimicrobial assays against strains from the phylogenetic tree may reveal sensitivity of other strains of clinical interest such as *Clostridium botulinum*.

The total synthesis of pediocin PA-1 allowed us to perform conformational studies by CD and NMR. In order to avoid interference due to Met31 oxidation and any conformational changes during NMR experiments with pediocin PA-1 **3c**, the M31L analog **5** was used, since substitution of Met31 with leucine neither decreased the antimicrobial activity nor modified the CD spectra (Fig. 4a). The three-dimensional structure of analog **5**, which possesses both of the disulfide bridges that occur in several type IIa bacteriocins, does not appear to differ substantially from those of other bacteriocins of the series. The mode of action of pediocin-like bacteriocins has been widely studied^47,48^. Indeed, these bacteriocins kill target cells by forming pores, disrupting the membrane integrity and function and causing dissipation of the proto-motive force, which is accompanied with depletion of intracellular ATP and leakage of intracellular ions and vital molecules. However a requisite is the interaction of the bacteriocin with the membrane-associated IIC and IID subunits of the manPTS system, thus forming a membrane-located complex. Several models of peptide/membrane interaction have been proposed for pediocin-like bacteriocins^26,29,66,67^. It is generally admitted that the helix is embedded in the membrane bilayer and the three-strand N-terminal β-sheet is located at the membrane/water interface together with the tryptophans, which are known to preferentially occur in the membrane/water interface. The structure of pediocin PA-1 M31L with tryptophans at positions 18 and 33 located at the two extremities of the helical region is compatible with this model. However, the structure predicted here can neither afford the detailed characteristics of the critical interaction between the peptide and the manPTS components nor determine if manPTS only acts as a docking molecule or contributes to the pore forming structure.

In summary, we report here a convenient and efficient procedure for the chemical synthesis of pediocin PA-1 and bioactive analogs thereof for further development to increase bacteriocin potency and stability and modify the spectrum of activity. The finding that linear pediocin PA-1 and replacement of the sensitive methionine with leucine does not affect antimicrobial activity and improves peptide stability will allow the production of this potent antimicrobial compound on a larger scale and in higher yields. This significant improvement in productivity will open the way for its use in the food industry and the veterinary or medical sectors. These modifications could be applied to a large array of class II and other bacteriocins containing methionine and/or disulfide bonds in order to increase their potential as substitutes for conventional antibiotics. With strong antimicrobial activities against major food-borne pathogens such as *Listeria* spp. and *Clostridium perfringens*, these pediocin PA-1 analogs have great potential in various sectors for prevention and treatment of infections. Studies are currently underway to evaluate their possible applications in the food industry and in human as well as veterinary medicine.

## Methods

### Materials and methods

All reagents and solvents were purchased from commercial suppliers and used without additional purification. Fmoc-amino acid derivatives and coupling reagents 2-(7-aza-1H-benzotriazole-1-yl)-1,1,3,3-tetramethyluronium hexafluorophosphate (HATU), 2-(1*H*-benzotriazol-1-yl)-1,1,3,3-tetramethyluronium hexafluorophosphate (HBTU) and 1-(mesitylene-2-sulfonyl)-3-nitro-1,2,4-triazole (MSNT) were purchased from Matrix innovations (Québec, QC, Canada), aminomethyl-ChemMatrix® resin (0.69 mmol/g) from PCAS Biomatrix Inc. (St-Jean-sur-Richelieu, QC, Canada), pseudoprolines from Gyros Protein Technologies (Tucson, AZ, USA) and 4-(4-hydroxymethyl-3-methoxyphenoxy)-butyric acid (HMPBA) from Chem-Impex (Wood Dale, IL, USA). Other reagents and solvents were purchased from Sigma-Aldrich.

LC-MS analyses were conducted on a Shimadzu Prominence LCMS-2020 system equipped with an electrospray ionization (ESI) probe using a Phenomenex Kinetex® EVO C18 column (4.6 × 100 mm, 2.6 µm XB-C_18_, 100 Å, 1.8 mL/min) with a 10.5 min gradient from water (0.1% HCOOH) and CH_3_CN (0.1% HCOOH) (CH_3_CN 10–100%) and detection at 220 nm and 254 nm. LC-MS/MS analyses and high-resolution mass spectrometry were performed on a Waters Synapt G2-Si (Quadrupole/TOF) with a Waters UPLC binary pump and FTN injector. The mass spectrometer was operated in high resolution mode and calibration done with a sodium formate (Sigma) solution and lock-mass correction using a leucine-enkephaline solution (Waters). Matrix-assisted laser desorption ionization time-of-flight (MALDI-TOF) mass spectrometry was performed on an AB SCIEX 4800 Plus MALDI-TOF/TOF instrument using alpha-cyano-4-hydroxycinnamic acid as matrix. The spectra were acquired using the 4000 Series Explorer Software (AbSciex, v 3.2.3). The PEAKS Studio software (Bioinformatics Solutions, v.7.0) was used for mass spectra analysis and *de novo* sequencing in combination of ProteinProspector v5.20.0 for manual sequencing.

### Peptide synthesis

Peptides were synthesized by standard solid-phase peptide synthesis (SPPS)^37^ on a Prelude peptide synthesizer from Gyros Protein Technologies (Tucson, AZ, USA) using HMPB-ChemMatrix® resin. First, the resin was prepared by swelling aminomethyl-ChemMatrix in DMF for 1 h followed by addition of HMPBA (3 equiv), HBTU (3 equiv), HOBt (3 equiv) and *N*-methylmorpholine (NMM) (6 equiv). After stirring the mixture for 3 h, the resin was washed with DMF (5×) and DCM (5×) and dried under vacuum. The C-terminal amino acid was anchored with the MSNT/*N*-methylimidazole method^68^. Briefly, Fmoc-Cys(Trt)-OH (5 equiv) was dissolved in anhydrous DCM with a minimal amount of anhydrous THF and the solution added to the resin swelled in anhydrous DCM. Afterward, MSNT (5 equiv) and *N*-methylimidazole (3.75 equiv) were dissolved in anhydrous DCM and the mixture incubated with the resin for 1 h under agitation. After washing the resin with DCM (5×) and DMF (5×), the peptide elongation was carried out by standard SPPS using the Fmoc/tBu strategy. Briefly, the Fmoc protecting group was removed from the resin by two 10 min treatments with 20% piperidine in DMF (v/v) and amino acid couplings were performed with Fmoc-Xaa-OH (3 equiv), HATU (3 equiv) and NMM (6 equiv) in DMF (2 × 30 min). Cleavage, side chain deprotection, and pseudoproline reopening were achieved by treating the resin with a mixture of TFA/TIS/H_2_O/phenol (90:5:2.5:2.5) for 1 h. After filtration, the product was precipitated with cold diethyl ether and the solid treated with TFA/TIS/H_2_O (95:2.5:2.5) for 2 h to ensure complete deprotection and pseudoproline reopening. The resulting peptide was precipitated with cold diethyl ether and the solid washed twice with diethyl ether to be dried under vacuum. The peptides were purified by RP-HPLC with a Shimadzu Prominence system on a Phenomenex Kinetex® EVO C18 column (250 × 21.2 mm, 300 Å, 5 µm) using 0.1% AcOH/H_2_O (A) and 0.1% AcOH/CH_3_CN (B), with a linear gradient of 5% to 50% (B) for 20 min at 14 mL/min and UV detection at 220 nm and 254 nm. The collected fractions were lyophilized to afford the desired peptide (**1**, **4**, or **6**) as a white powder using an SC250EXP Speedvac Concentrator (ThermoFisher Scientific, Waltham, MA, USA).

### Disulfide bond formation

The purified linear peptide (**1** or **4**) was dissolved in CH_3_CN/H_2_O (1:1) at a concentration of 1 mg/mL and cyclized by adding *N*-chlorosuccinimide (2.4 equiv)^44^. After stirring for 30 min, the product was lyophilized and purified by RP-HPLC as described above to yield the cyclic peptide (**2a**, **2b**, **2c** or **5**).

### Selective methionine reduction

Reduction of the oxidized Met was carried out in a solution of TFA, β-mercaptoethanol and anisole (95:2.5:2.5) at 1 mg/mL in the presence of TBAB (30 equiv) for 5 min at room temperature^46^. After precipitation and washing with cold diethyl ether, the resulting product (**3a**, **3b** or **3c**) was analyzed by RP-HPLC and MS.

### Antimicrobial assays

The antibacterial activity of the synthetic peptides was assessed in radial diffusion assays^69^. ATCC strains were obtained from the American Type Culture Collection (Rockville, MD, USA), *L. monocytogenes* LSD530 from the Canadian Food Inspection Agency, (Laboratory Services Division, Ottawa, ON, Canada), *P. acidilactici* UL5 from STELA Dairy Research Center culture collection (Université Laval, Québec, QC, Canada), *L. ivanovii* HPB28 from Public Health Agency of Canada (Ottawa, ON, Canada), *C. perfringens* strains AAC-122 and AAC-123 from Agriculture and Agri-Food Canada (Ottawa, ON, Canada), *B. cereus* LSPQ2872 from the Laboratoire de santé publique du Québec (Québec, QC, Canada), *L. salivarius* PIB16 from pig gastrointestinal tract, sequenced from 16S rRNA^70^ and *S. thermophilus* RBL 18 FYE 41 from Lacto-labo Danisco, (Vienne, France). Samples (75 µL) containing 1 mg/mL of purified peptide were placed in wells in Man-Rogosa-Sharpe (MRS) (Oxoid, Nepean, ON, Canada), reinforced clostridium medium (RCM) (Nutri-Bact, Terrebonne, QC, Canada), brain heart infusion (BHI), Mueller-Hinton (MH) or tryptic soy broth (Difco Laboratories, Sparks, MD, USA) supplemented with 0.6% yeast extract (w/v) (TSBY) soft agar (0.75% w/v) and seeded with the appropriate strain (e.g. MRS for *P. acidilactici, *TSBY for *Listeria* spp.) as detailed in Tables 3 and S1. The Petri plates (100 × 15 mm, VWR, Radnor, PA, USA) were incubated at 37 °C for 18 h and antibacterial activity was observed as a halo of inhibition in the bacterial lawn formed around the sample. Nisin and microcin J25 in culture supernatants were used as positive controls. They were obtained from *Lactococcus lactis* subsp. lactis ATCC 11454 cultivated in MRS broth and *Escherichia coli* MC4100 pTUC202 in M69 broth, respectively after 18 h at 37 °C. All strains were reactivated from 20% glycerol stock at −80 °C and sub-cultured at least three times at 24-h intervals before use. Pictures were taken with ChemiDoc XRS (Bio-Rad, Hercules, CA, USA).

Minimal inhibitory concentrations (MIC) were determined using polystyrene micro-assay plates (96-well 353072 Falcon, Corning, NY, USA)^71^. Briefly, micro-plates loaded with twofold serial dilutions of each of the synthetic peptides (starting at 250 μM) in the appropriate culture medium were seeded with log-phase culture of target strain diluted in the same culture medium to 0.5–1.0 × 10^6^ cfu ml^−1^ (approximately 1 × 10^4^ cfu per well). Micro-plates were then incubated at 37 °C for 18 h and absorbance at 595 nm was measured hourly using an Infinite ® F200 PRO photometer (Tecan US inc., Durham, NC, USA). MIC values were expressed in nM and correspond to the lowest concentrations that inhibited the growth of the target organisms after 18 h. MIC values are reported as means of two independent experiments in duplicate.

### Phylogenetic analysis

BLAST analysis for manPTS IID subunit protein was performed using the blastp command of the NCBI non-redundant protein database (BLASTP version 2.6.1+) with the standard parameters^72^.

### Circular dichroism

Peptides were dissolved in 0.1% TFA/H_2_O (2.5 mg/mL) and diluted to 0.1 mM in aqueous TFE solutions (0, 25, 50, 75 or 90% TFE in H_2_O). For the study in phospholipid vesicles, a lipid/peptide ratio of 100:1 was used. Briefly, DMPC or DMPG was dissolved in MeOH and the mixture dried with a stream of nitrogen. Peptides were dissolved in phosphate buffer (20 mM, pH 7.4) (1 mg/mL) and added to the dried phospholipid films. After hydration of the lipids, the mixtures were sonicated with a Branson 2510 bath sonicator (Branson Ultrasonics, Danbury, CT, USA) for 5 min or until a clear solution was obtained. CD measurements of the peptides in aqueous TFE solutions and in phospholipid vesicles were performed with a Jasco J-815 spectrometer (Aviv Instruments, Lakewood, NJ, USA). The spectra were recorded at 25 °C in the 190–260 nm wavelength range at 0.1 nm intervals in a cuvette with a 0.1 mm path length. For each spectrum, 10 scans were averaged and smoothed by the J720/98 system program (Version 120C). CD data were expressed as mean residue molar ellipticity [*θ*] given in deg cm^2^ dmol^−1^, plotted against wavelength (nm) and analyzed using the CONTIN algorithm included in the CDPro analysis software^73^.

### NMR spectroscopy

Samples were prepared using 2 mg of pediocin PA-1 M31L **5** dissolved in 300 µL of 0.1% TFA in water plus 300 µL of 98% TFE-d2 in a Wilmad 3 mm tube (Rototec Spintec). Experiments were performed on a Bruker Avance III 600 MHz spectrometer equipped with a cryoprobe. ^1^H chemical shifts were referenced to the TFE resonance taken at 3.88 ppm downfield from TMS. Temperature effects on the structure were surveyed by recording ^1^H spectra at 288, 298, 303, 308, 313 K with water suppression using sculpting with gradients. For sequential assignment, TOCSY and NOESY experiments were recorded at 313 K in the phase-sensitive mode using States-TPPI. TOCSY and NOESY spectra were recorded with mixing times of 80 ms and 300 ms using 16 and 72 scans, respectively. Water suppression was achieved using excitation sculpting^74,75^. All spectra were processed with Bruker TOPSPIN 3.5.

### Modeling and structure simulations

Structures were obtained using chemical shifts (Table S3) as input for the Chemical Shift Rosetta software (Rosetta version 3.8, CS-Rosetta Toolkit version 3.3)^76^. Disulfide bridge constraints were added for C9–C14 and C24–C44. A total of 40,000 structures were generated. Complementary calculations were also performed in parallel with the two other disulfide bond pairings (C9–C24/C14–C44 and C9–C44/C14–C24) using a total of 5000 structures. The quality of the lowest energy structure was evaluated using Molprobity program v4.4. The aligned α-helix was calculated using SuperPose 1.0 software.

## Electronic supplementary material

Supplementary Information
