# Supplementary material for: Synthesis, antimicrobial activity and conformational analysis of the class IIa bacteriocin pediocin PA-1 and analogs thereof
Source: Sci Rep. 2018 Jun 13;8:9029. doi: 10.1038/s41598-018-27225-3 (PMC5998028; doi:10.1038/s41598-018-27225-3)
Supplement: Supplementary file 1 — Supplementary Information [file 41598_2018_27225_MOESM1_ESM.pdf]

Supporting Information

Synthesis, antimicrobial activity and conformational analysis of the class IIa bacteriocin pediocin PA-1 and analogs thereof

François Bédard[1,2], Riadh Hammami[2,†], Séverine Zirah[3], Sylvie Rebuffat[3], Ismail Fliss[2], Eric Biron*[,1]

[1]*Faculté de Pharmacie, Université Laval and Laboratoire de chimie médicinale, Centre de recherche du CHU de Québec, 2705 Boulevard Laurier, Québec, Québec G1V 0A6, Canada*

[2]*STELA Dairy Research Centre, Institute of Nutrition and Functional Foods, Université Laval, Québec, Québec G1V 0A6, Canada*

[3]*Molécules de Communication et Adaptation des Microorganismes (MCAM, UMR 7245), Muséum national d'Histoire Naturelle, Sorbonne Universités, CNRS, CP 54, 57 rue Cuvier, 75005, Paris, France*

[†]*Present address: School of Nutrition Sciences, University of Ottawa, Ottawa, ON, Canada, K1N 6N5*

*Corresponding author: eric.biron@pha.ulaval.ca

Figure S1. HPLC profiles (λ = 220 nm) and ESI-MS spectra of purified linear pediocin PA-1 **1** and crude oxidized pediocin PA-1 **2a-c**.

KYYGNGVTCGKHSCSVDWGKATTCIINNGAMAWATGGHQGNHKC **1**

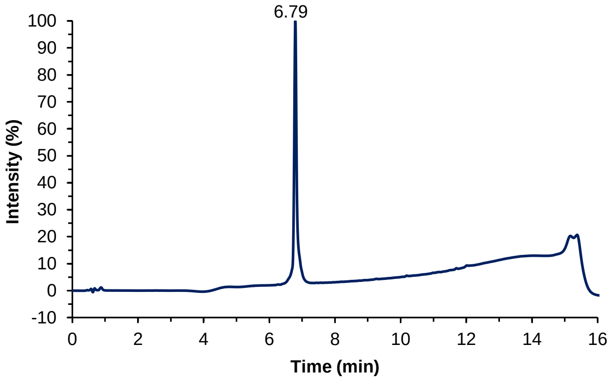

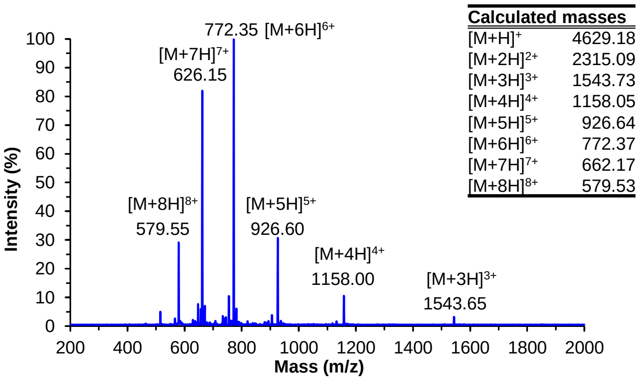

KYYGNGVTCGKHSCSVDWGKATTCIINNGAM(O)AWATGGHQGNHKC **2a**

+

KYYGNGVTCGKHSCSVDWGKATTCIINNGAM(O)AWATGGHQGNHKC **2b**

+

KYYGNGVTCGKHSCSVDWGKATTCIINNGAM(O)AWATGGHQGNHKC **2c**

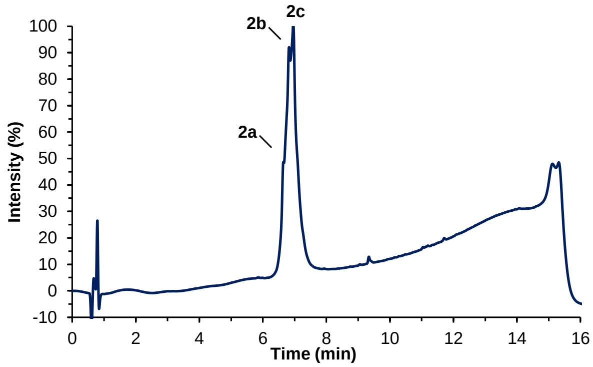

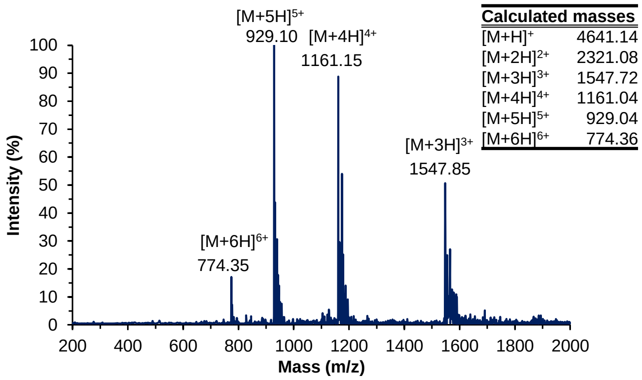

Figure S2. HPLC profiles (λ = 220 nm) and ESI-MS spectra of purified oxidized pediocin PA-1 analogs **2a**, **2b** and **2c**.

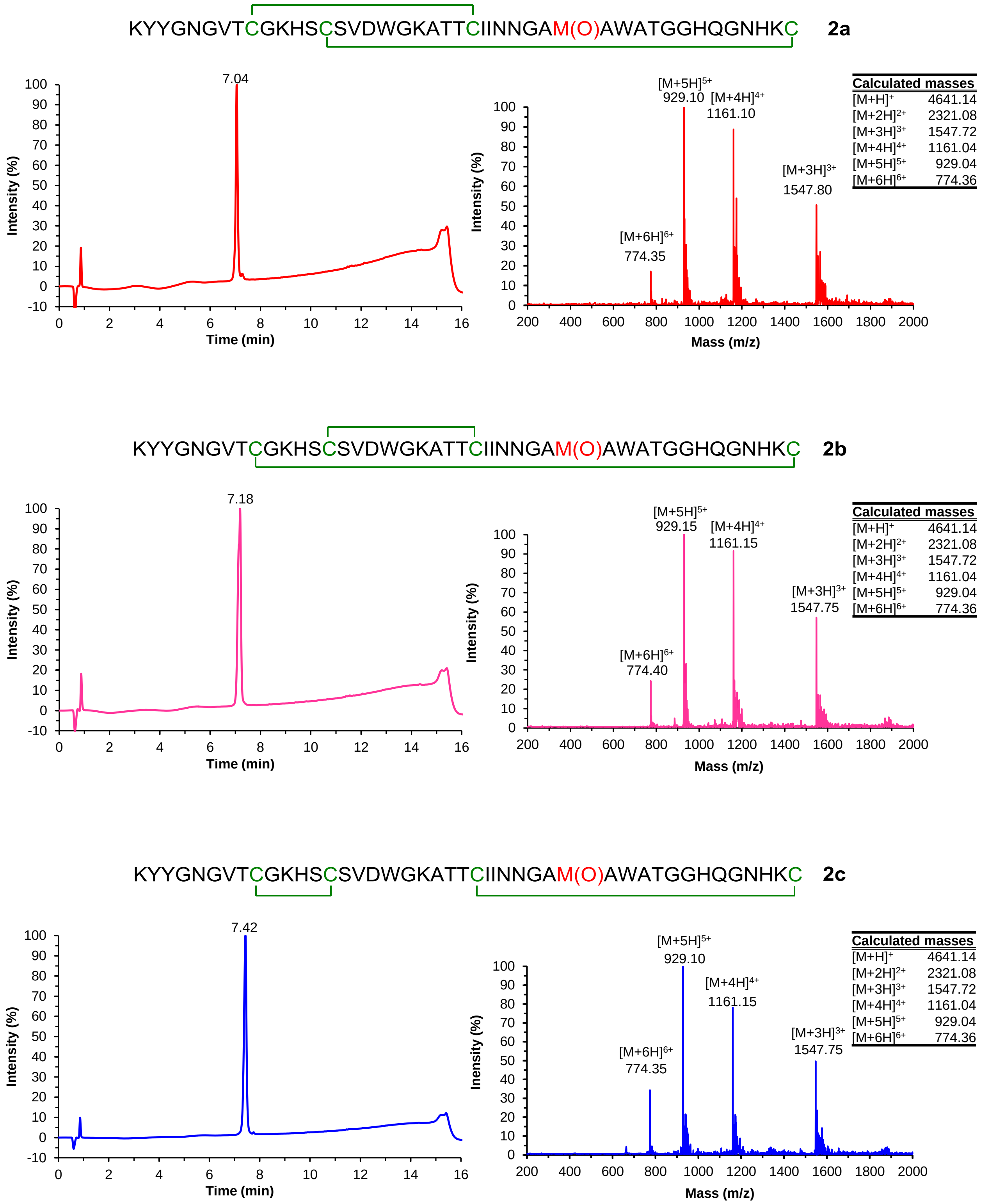

Figure S3. HPLC profiles (λ = 220 nm) and ESI-MS spectra of purified pediocin PA-1 **3c** and its analogs **3a** and **3b**.

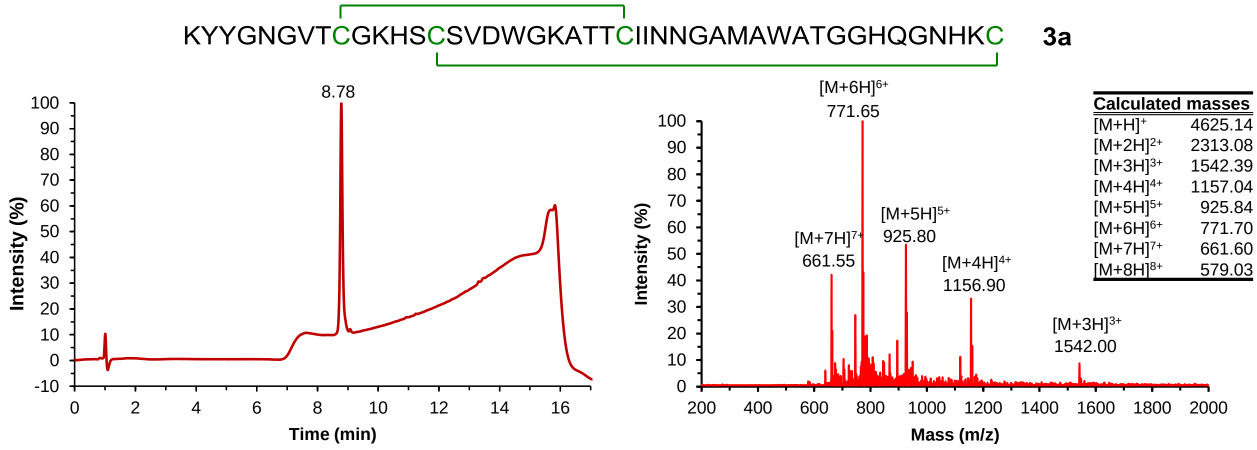

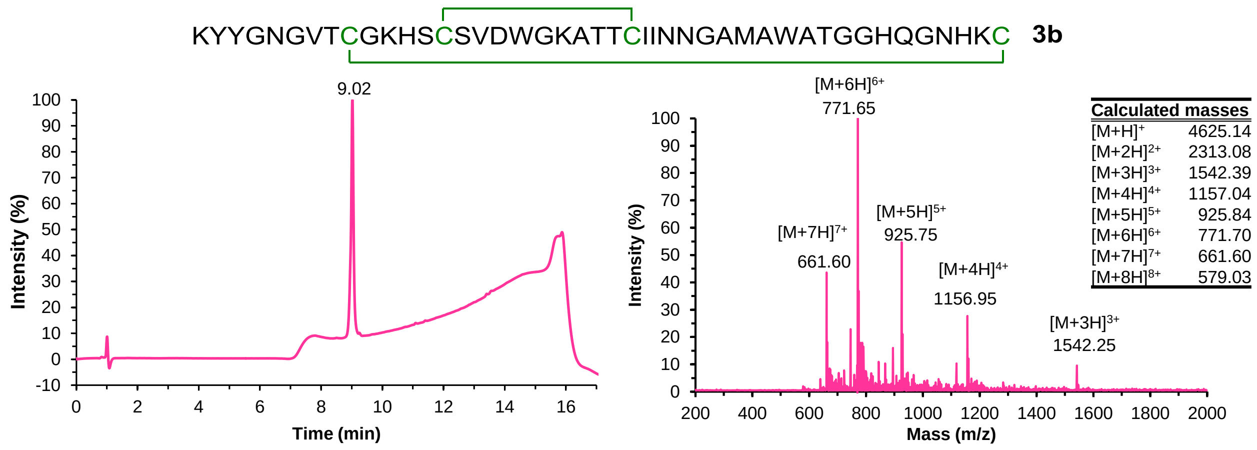

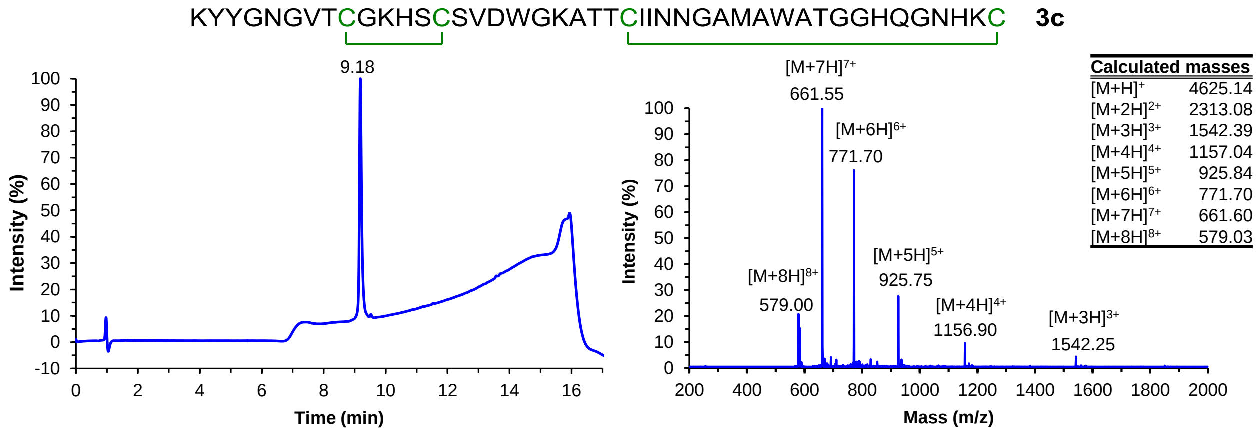

Figure S4. MALDI TOF MS/MS spectra of fragments obtained after treatment of *S*-acetamidomethylated synthetic pediocin-PA-1 **3c** with trypsin.

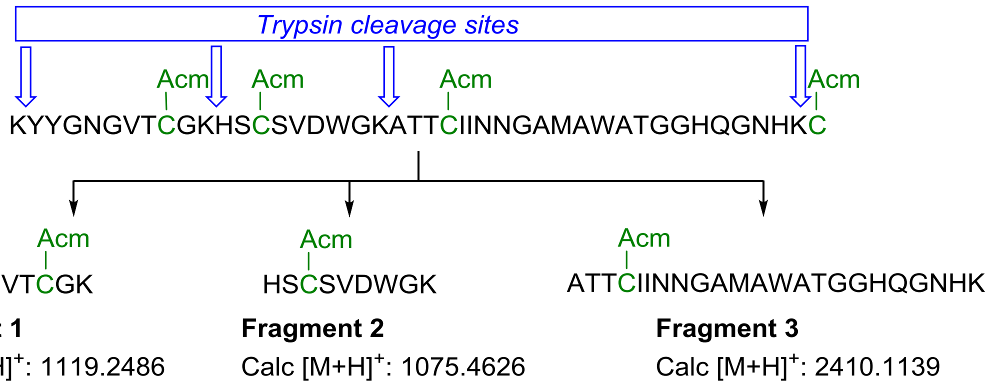

A) Fragment 1: MS/MS spectrum of molecular ion at m/z 1119.45

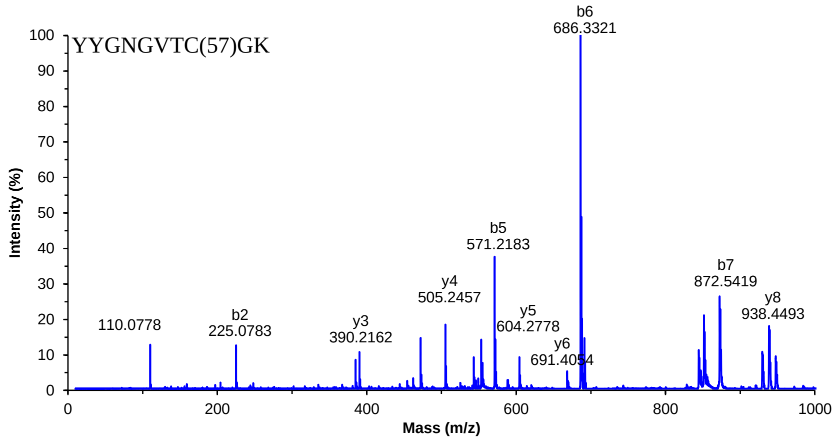

Calculated main sequence ions for fragment 1

b		Amino acid		*y*
---	1	H	9	---
225.0982	2	S	8	938.3822
385.1074	3	C(57)	7	851.3502
472.1394	4	S	6	691.3410
571.2078	5	V	5	604.3089
686.2348	6	D	4	505.2405
872.3141	7	W	3	390.2136
929.3356	8	G	2	204.1343
---	9	K	1	147.1128

B) Fragment 2: MS/MS spectrum of molecular ion at m/z 1075.42

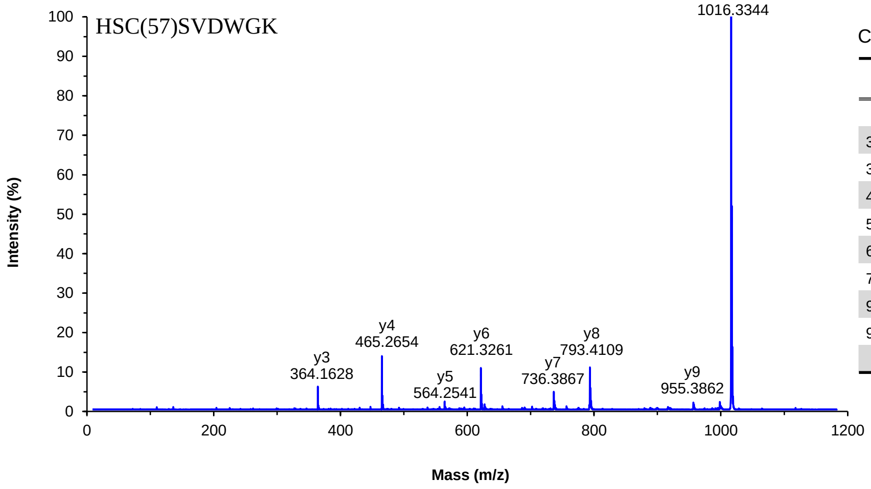

Calculated main sequence ions for fragment 2

b		Amino acid		*y*
---	1	Y	10	---
327.1339	2	Y	9	955.4087
384.1554	3	G	8	792.3454
498.1983	4	N	7	735.3239
555.2198	5	G	6	621.2810
654.2882	6	V	5	564.2595
755.3359	7	T	4	465.1911
915.3451	8	C(57)	3	364.1435
972.3665	9	G	2	204.1343
---	10	K	1	147.1128

Figure S4. (continued)

C) Fragment 3: MS/MS spectrum of molecular ion at m/z 2410.42

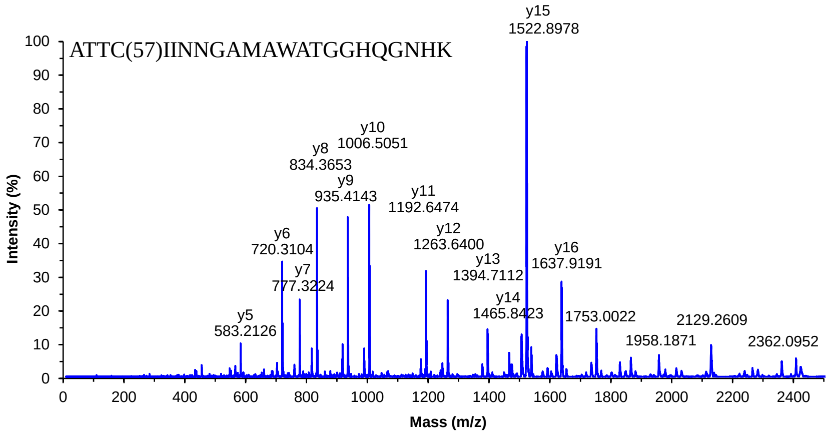

Calculated main sequence ions for fragment 3

b		Amino acid		*y*
---	1	A	23	---
173.0921	2	T	22	2339.0553
274.1397	3	T	21	2238.0077
434.1489	4	C(57)	20	2136.9600
547.2330	5	I	19	1976.9508
660.3171	6	I	18	1863.8667
774.3600	7	N	17	1750.7827
888.4029	8	N	16	1636.7397
945.4244	9	G	15	1522.6968
1016.4615	10	A	14	1465.6753
1147.5020	11	M	13	1394.6382
1218.5391	12	A	12	1263.5977
1404.6184	13	W	11	1192.5606
1475.6555	14	A	10	1006.4813
1576.7032	15	T	9	935.4442
1633.7247	16	G	8	834.3965
1690.7461	17	G	7	777.3751
1827.8050	18	H	6	720.3536
1955.8636	19	Q	5	583.2947
2012.8851	20	G	4	455.2361
2126.9280	21	N	3	398.2146
2263.9869	22	H	2	284.1717
---	23	K	1	147.1128

Figure S5. LC-MS/MS analysis of crude synthetic pediocin-PA-1 **3**.

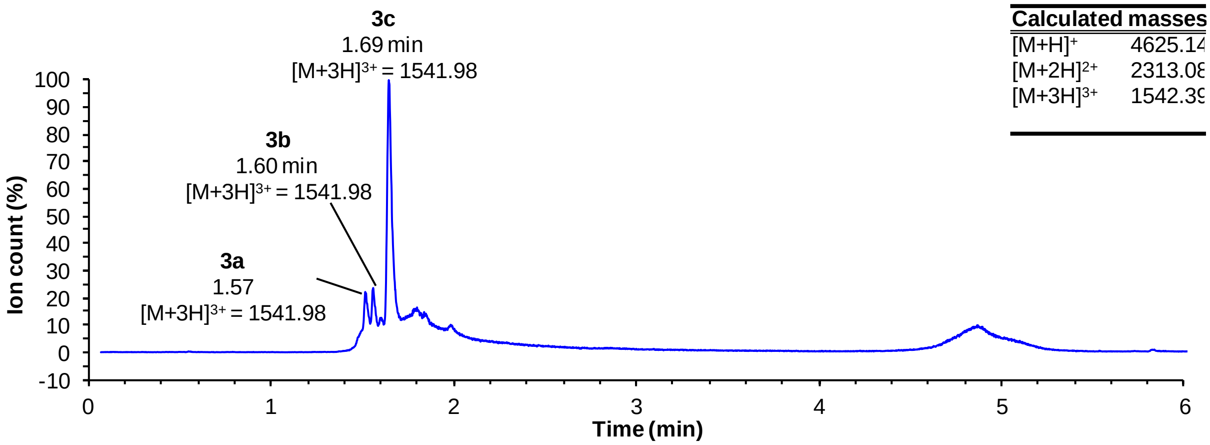

MS/MS spectrum of the 1.69 min peak (molecular ion $[M+3H]^{3+}$ at m/z 1541.98)

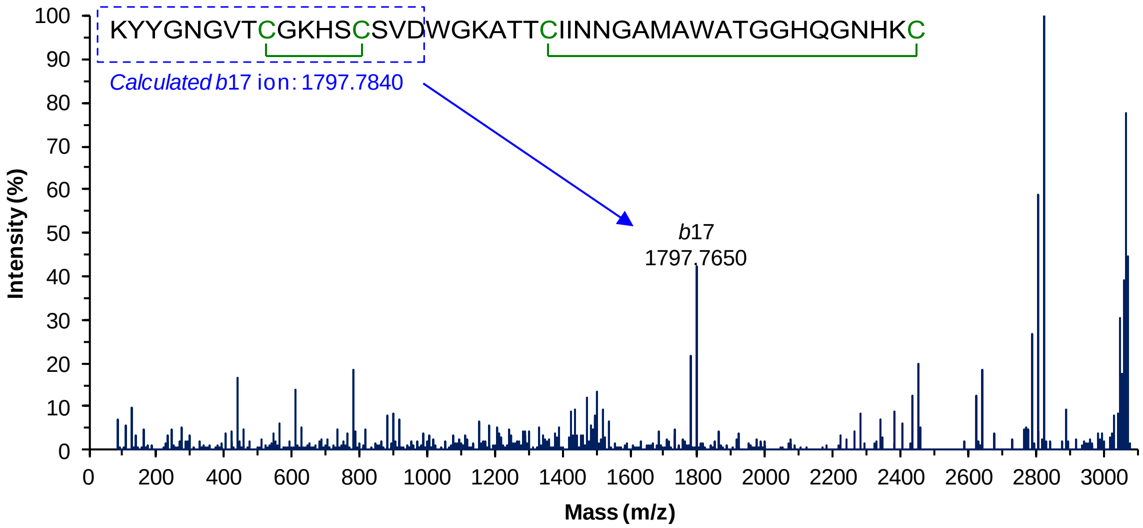

The *b*17 ion is only possible for **3c**. This fragment cannot be generated with **3a** or **3b**.

Figure S5. (continued)

MS/MS spectrum of the 1.57 min peak (molecular ion $[M+3H]^{3+}$ at m/z 1541.98)

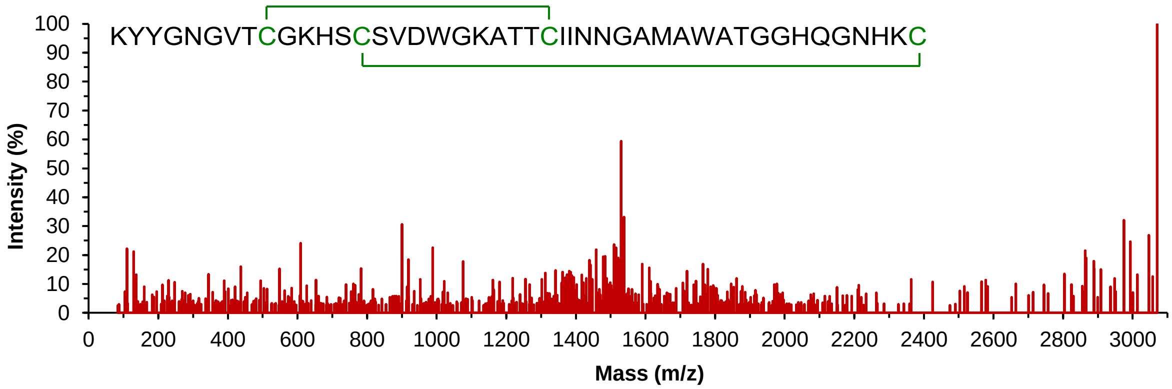

MS/MS spectrum of the 1.60 min peak (molecular ion $[M+3H]^{3+}$ at m/z 1541.98)

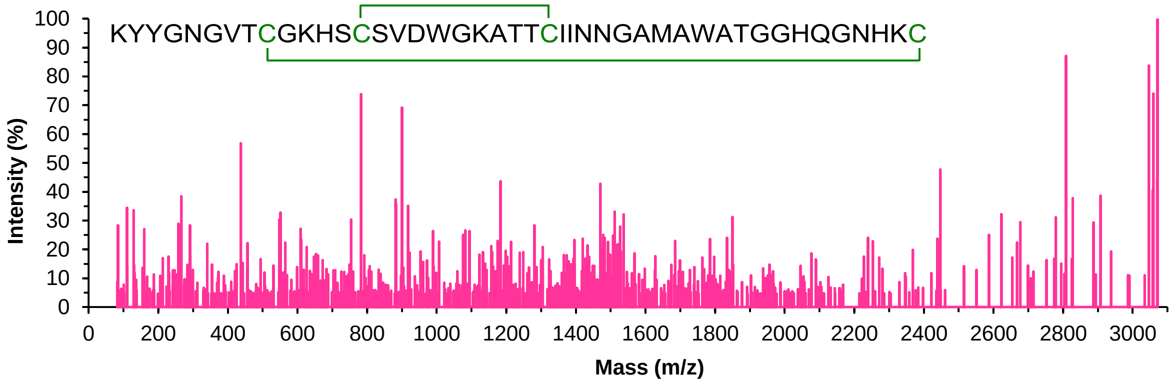

Figure S6. HPLC profiles (λ = 220 nm) and ESI-MS spectra of purified pediocin PA-1 analogs **4**, **5**, and **6**.

KYYGNGVTCGKHSCSVDWGKATTCIINNGALAWATGGHQGNHKC **4**

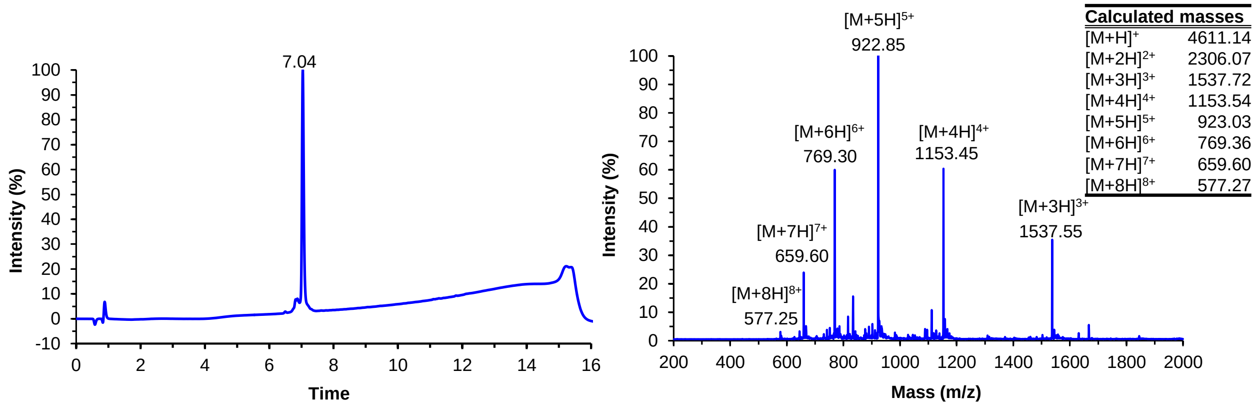

KYYGNGVTCGKHSCSVDWGKATTCIINNGALAWATGGHQGNHKC **5**

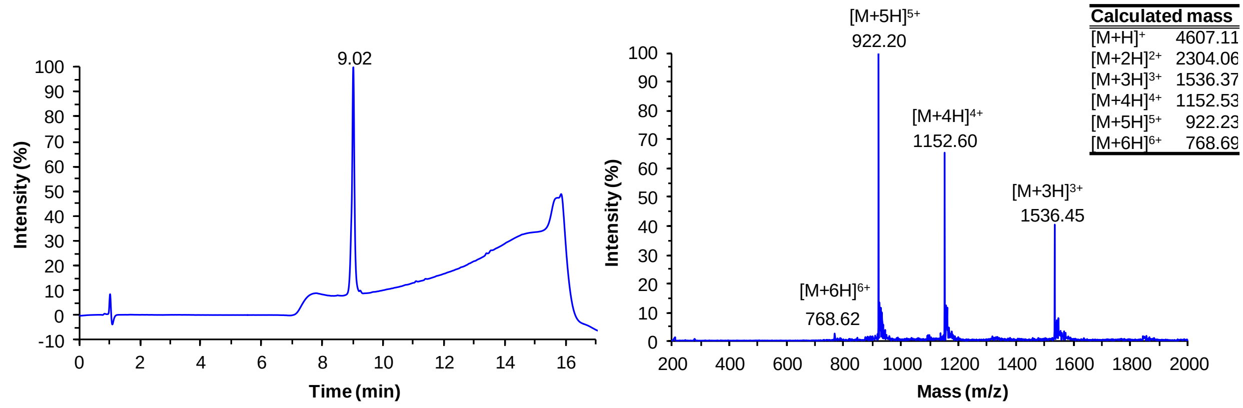

KYYGNGVTAGKHSASVDWGKATTAIINNGAMAWATGGHQGNHKA **6**

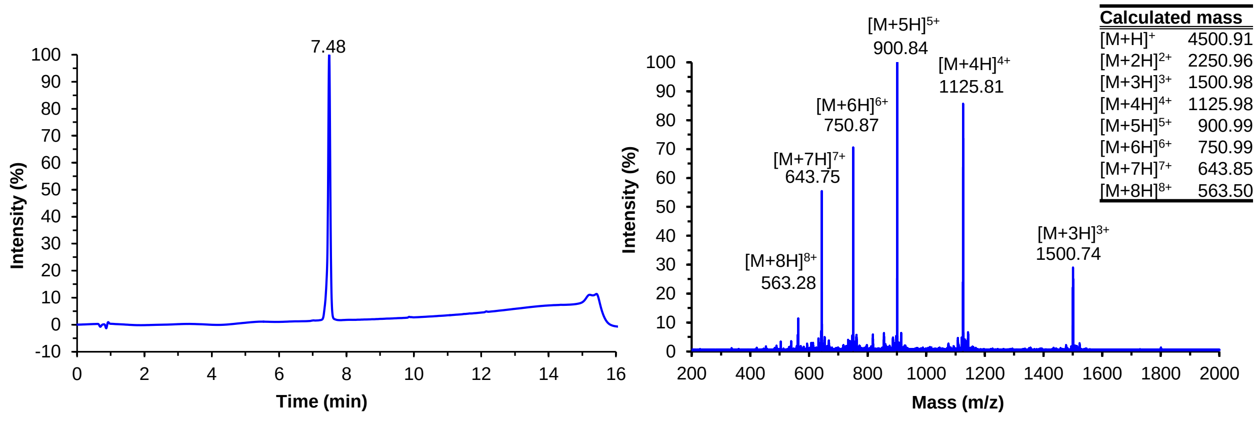

Figure S7. Phylogenetic tree obtained from protein Blast with manPTS IID sequence of targeted pathogen L. monocytogenes (ref|WP_003721724.1).

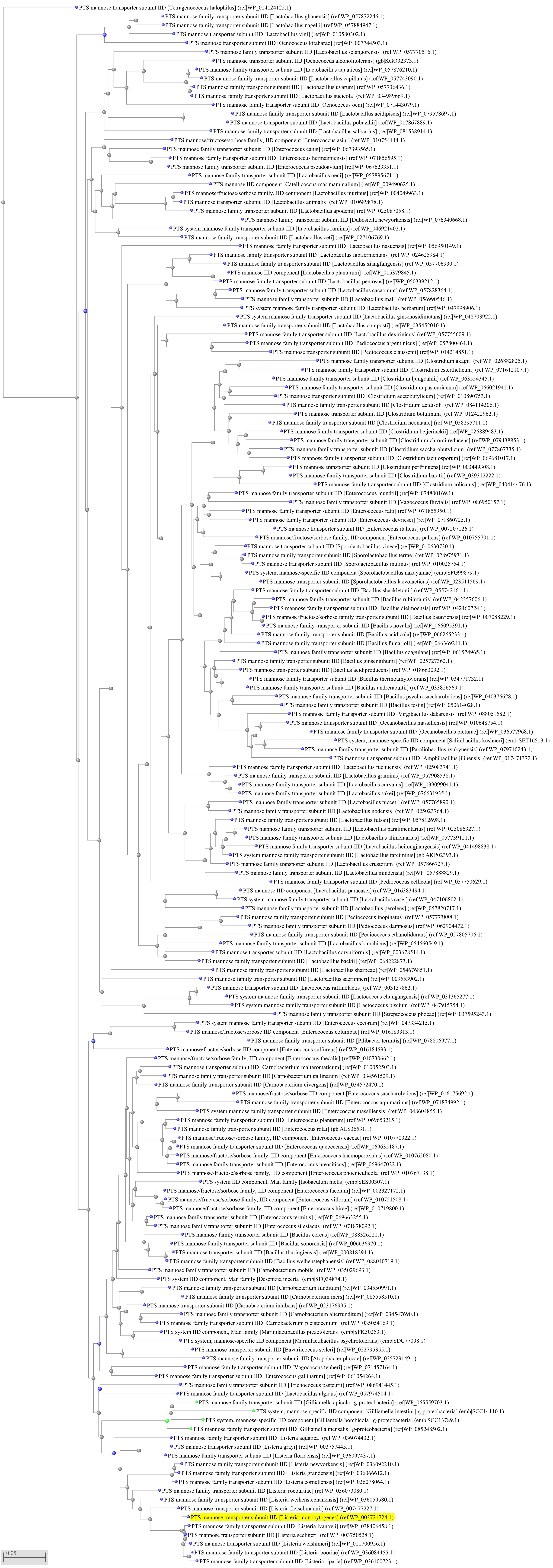

Figure S8. Agar diffusion assay of the linear pediocin PA-1 M31L analog **4** against sensitive strains. Inhibition diameter for analog **4**, supernatant of *Pediococcus acidilactici* UL5, and supernatant of *Lactococcus lactis* subsp. lactis ATCC 11454, respectively: A) 28 mm, 18 mm, 12 mm; B) 35 mm, 28 mm, 16 mm; C) 32 mm, 23 mm, 25 mm; D) 35 mm, 21 mm, 14 mm; E) 23 mm, 17 mm, 18 mm; F) 25 mm, 19 mm, 0 mm.

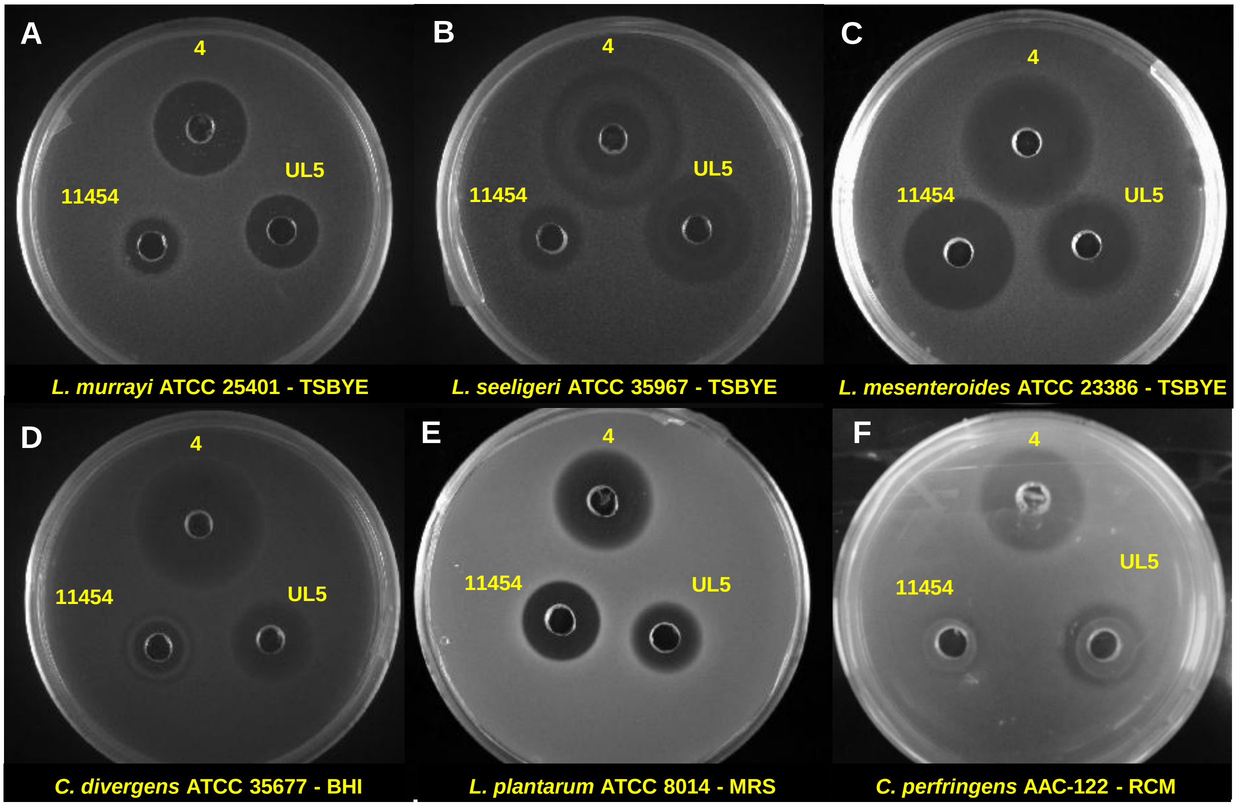

**C. perfringens* picture was taken with Sony FDR-X3000. All others pictures as described in material and methods from article; ChemiDoc XRS (Bio-Rad, Hercules, CA, USA).

Figure S9. Circular dichroism spectra of analogs **1**, **2c**, **3a-c** and **6** in water and aqueous TFE solutions (% of TFE in water is shown).

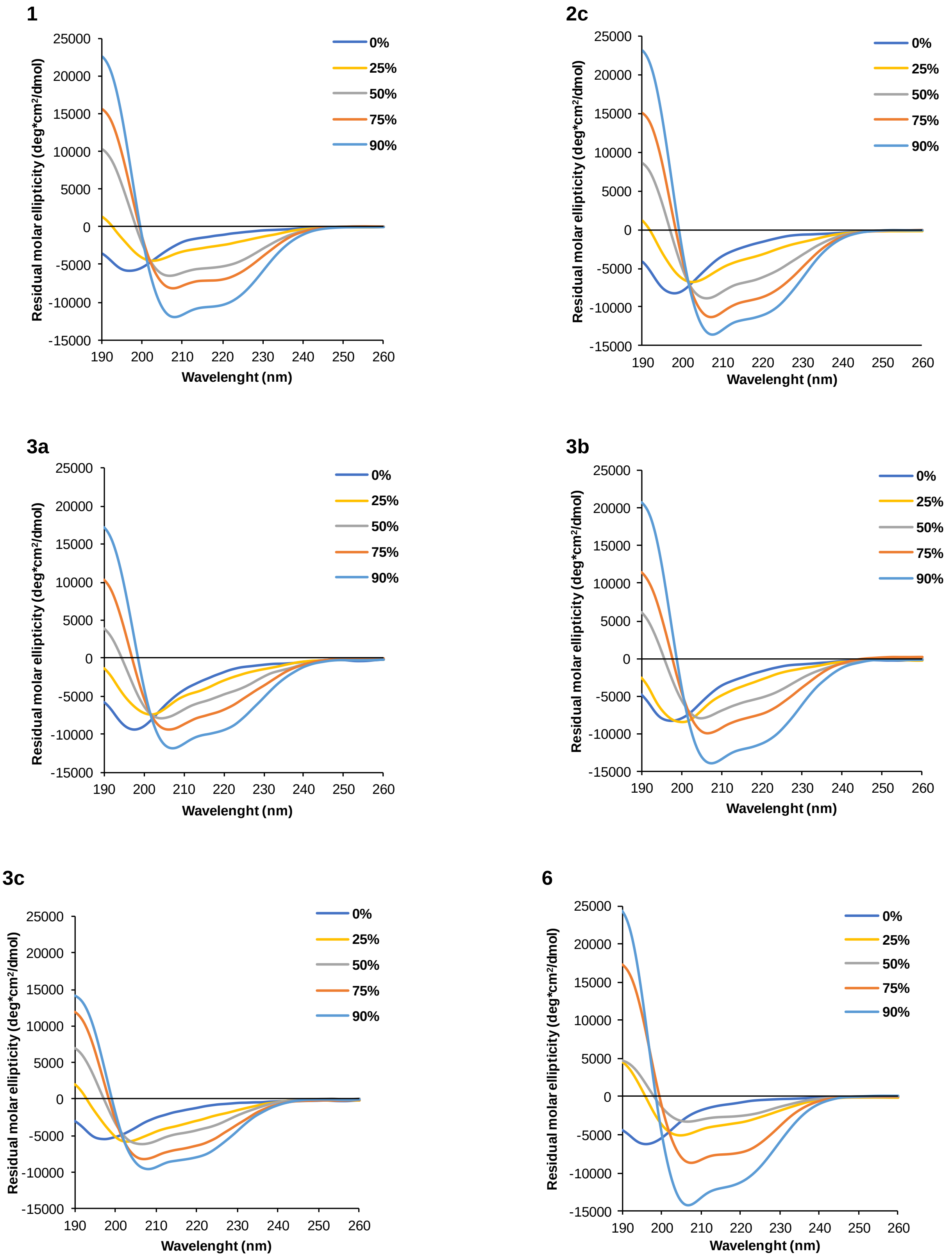

Figure S10. ^{1}H NMR spectrum of pediocin PA-1 M31L (analog **5**) at 313 K in H_2O/TFE-d2 (1:1) containing 0.1% TFA.

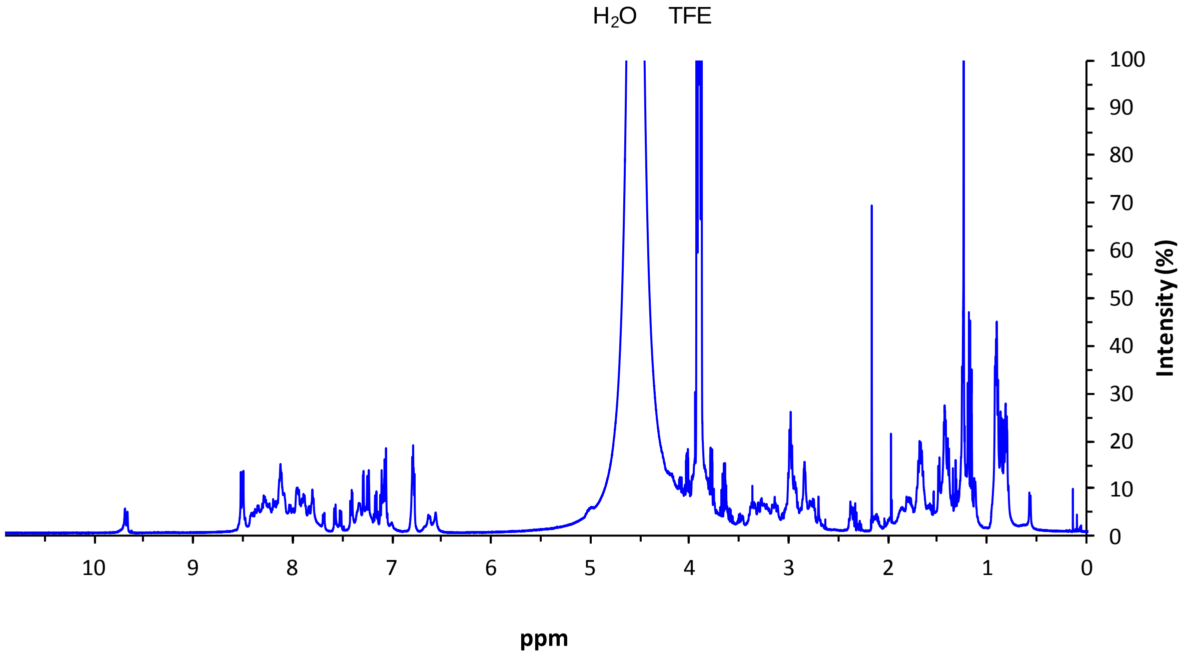

Figure S11. Superposed 2D NMR TOCSY (black) and NOESY (blue) spectra of pediocin PA-1 M31L (analog **5**) at 313 K in H_2O/TFE-d2 (1:1) containing 0.1% TFA.

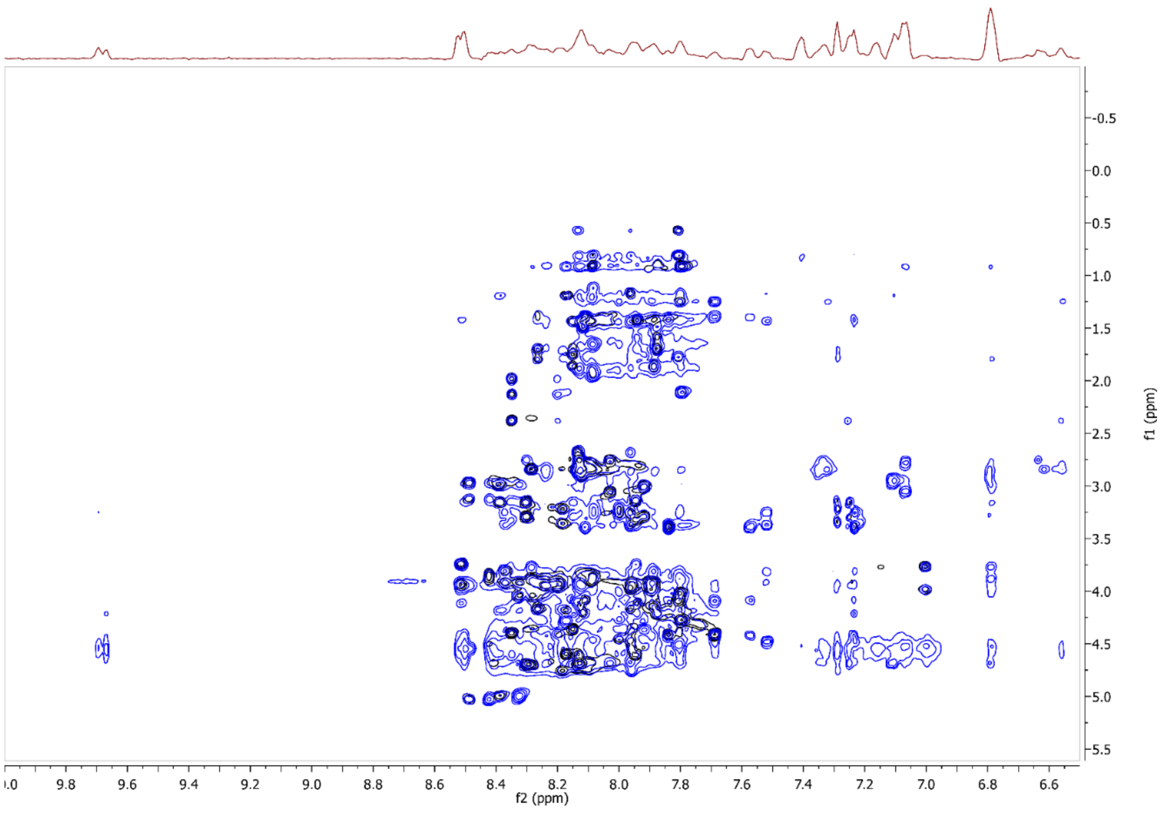

Figure S12. Energy/RMSD plots showing the convergence of the 5000 lowest RMSD structures of pediocin PA-1 M31L (analog **5**).

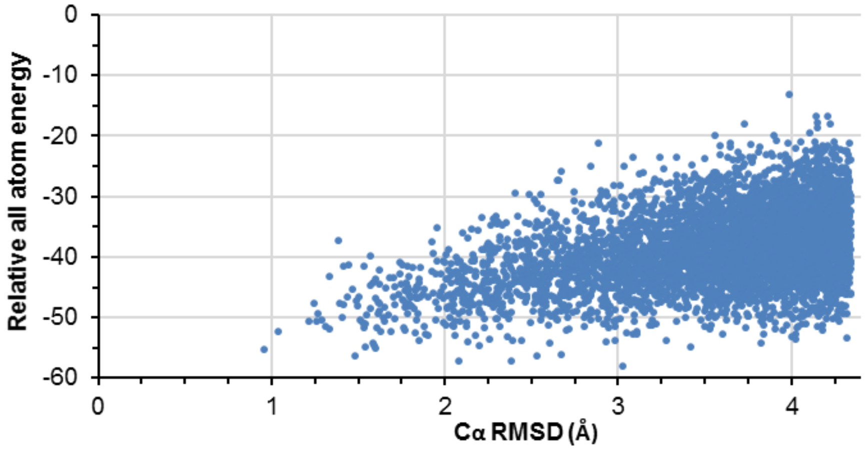

Figure S13. Energy/RMSD plots obtained for the 5000 structures calculated using pediocin PA-1 M31L (analog **5**) chemical shifts and the two non-natural disulfide bond pairings A) Cys_9-Cys_{24} and B) Cys_{14}-Cys_{44}.

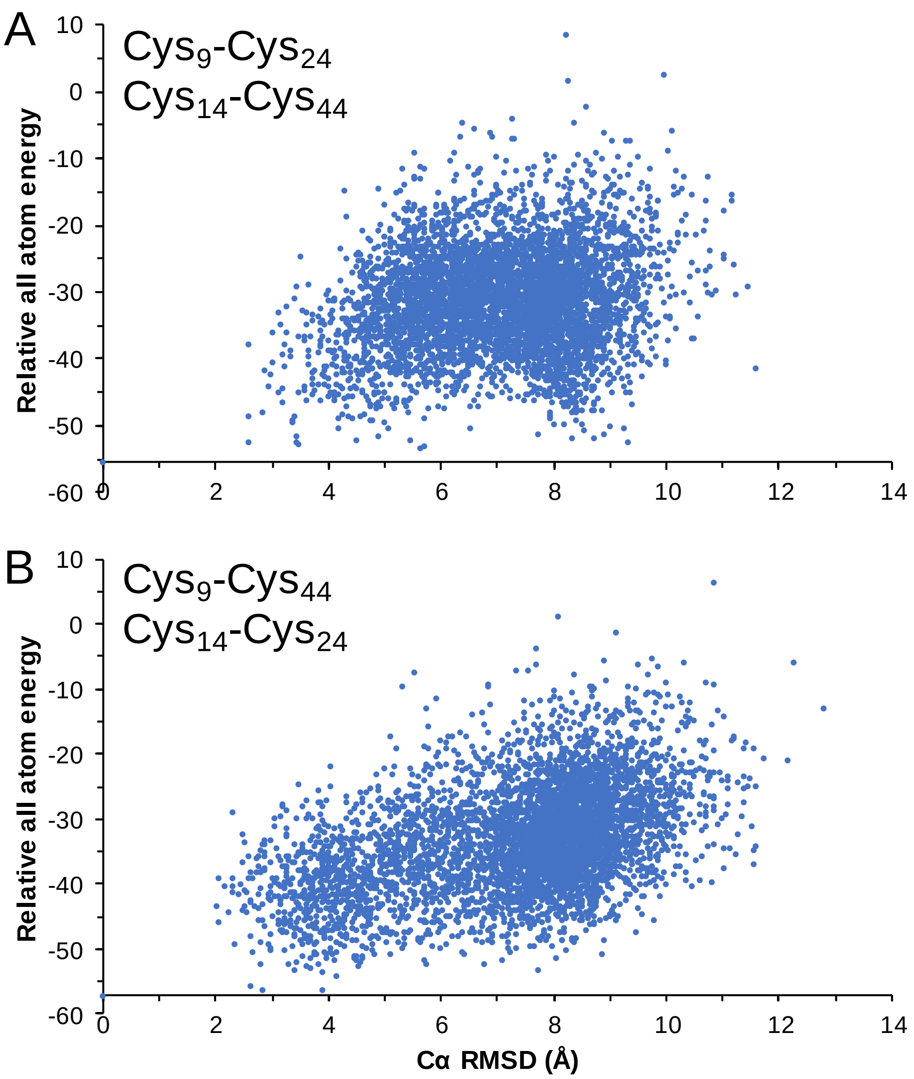

Figure S14. Chemical shift deviation (CSD) of the Hα of pediocin PA-1 M31L (analog **5**) in H_2O/TFE-d2 (1:1).

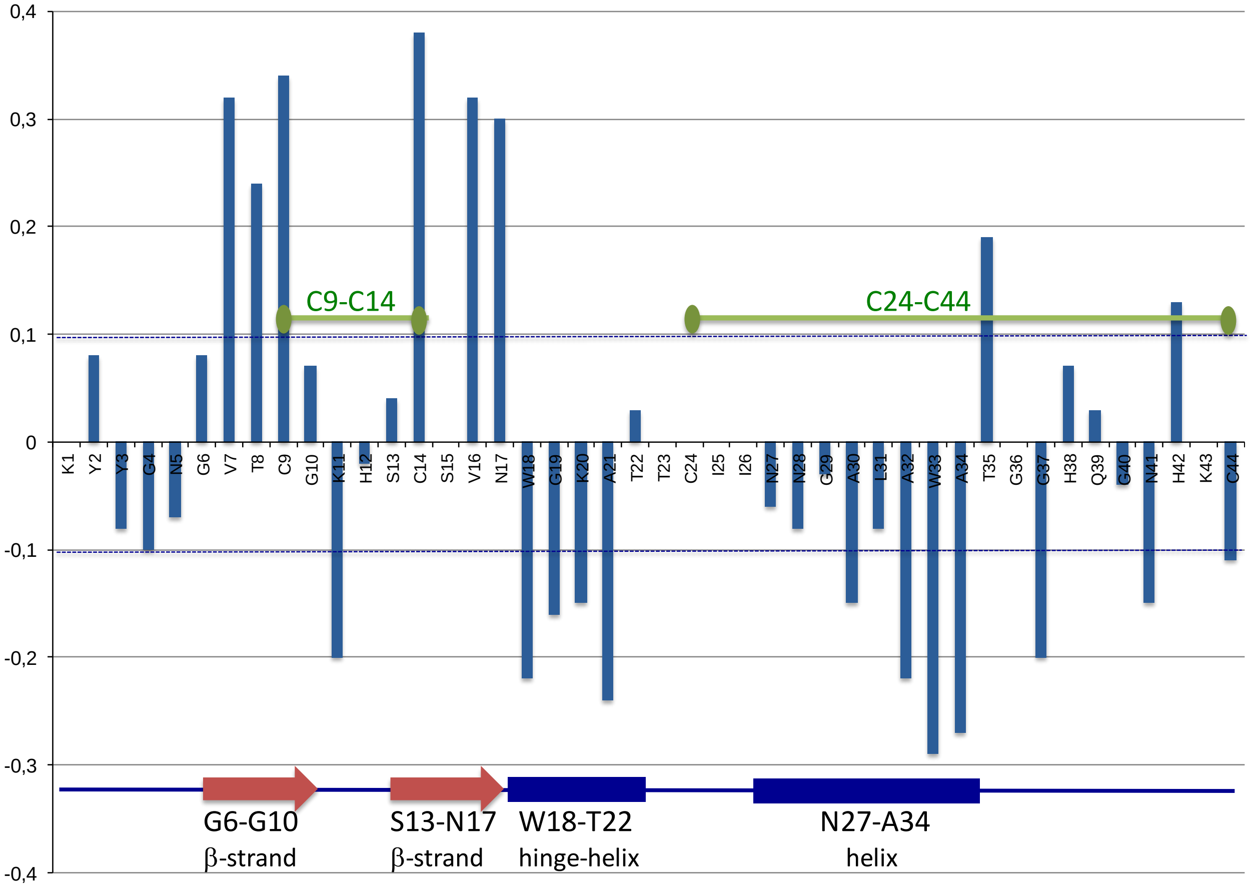

Figure S15. Ramachadran plot (Molprobity) for 10 lowest energy structures of pediocin PA-1 M31L (analog **5**) obtained from CS-Rosetta. Structure verification showed 91.2% most favoured, 8.8% additionally allowed, 0.0% generously allowed, 0.0% disallowed regions. A) general, B) isoleucine and valine, C) glycine.

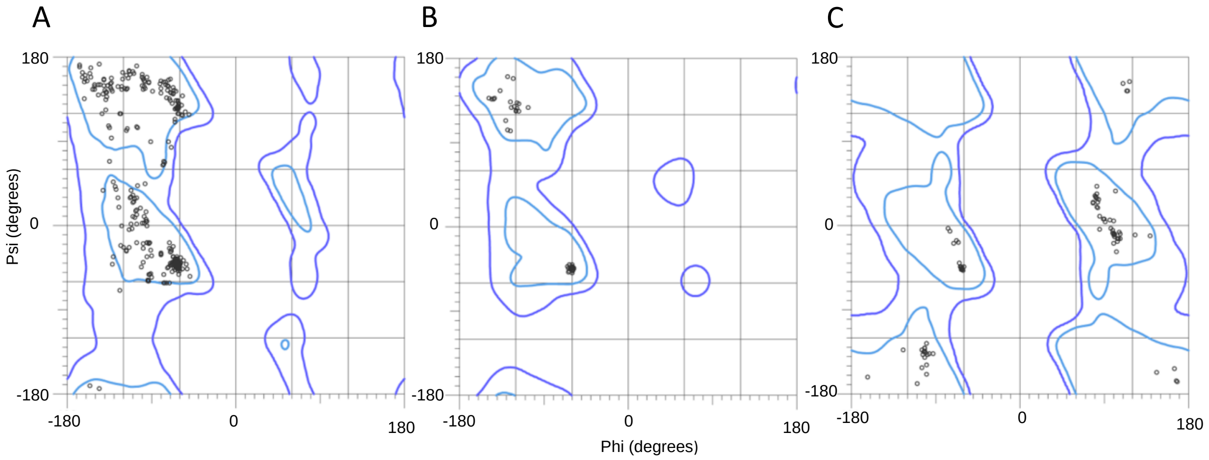

Figure S16. Cα RMSD per residue from α-helix aligned structures for pediocin PA-1 M31L (analog **5**).

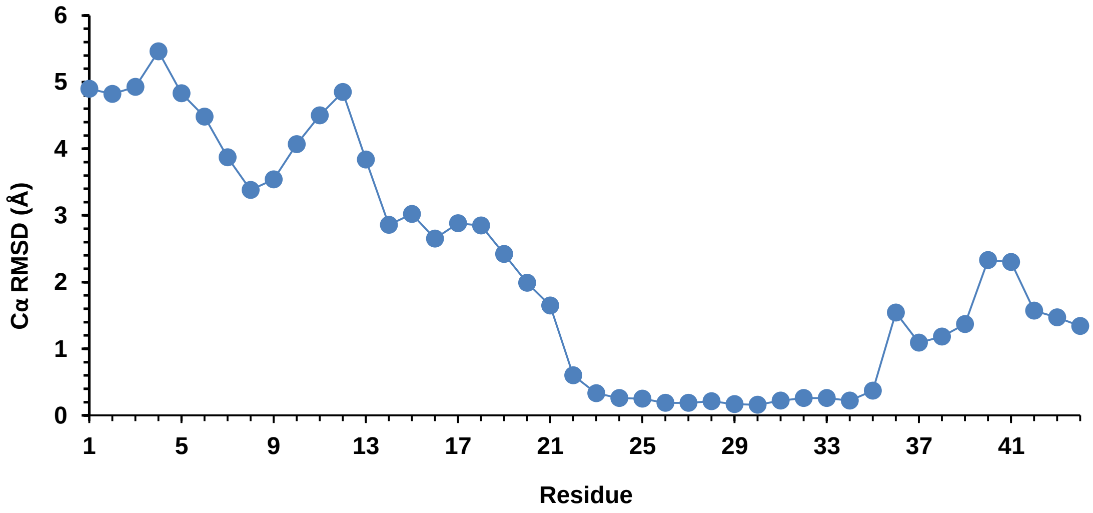

Table S1. Antimicrobial assays of synthetic analog **4** tested against strains that showed low or very low sensitivity.

Strains	MIC (nM)	Medium	Reference
Lactobacillus salivarius PIB16	38752	MRS	Pr I. Fliss*
Bifidobacterium animalis ATCC 27536	n.a.	RCM	ATCC
Enteroccocus faecalis ATCC 27275	n.a.	BHI	ATCC
Bacillus coagulans ATCC 7050	n.a.	MH	ATCC
Bacillus subtilis ATCC 6633	n.a.	MH	ATCC
Bacillus cereus LSPQ 2872	n.a.	MH	LSPQ
Escherichia coli ATCC 25922	n.a.	MH	ATCC
Staphylococcus aureus ATCC 25923	n.a.	MH	ATCC
Staphylococcus aureus ATCC 29213	n.a.	MH	ATCC
Staphylococcus aureus ATCC 6538	n.a.	MH	ATCC
Enterococcus hirae ATCC 10541	n.a.	BHI	ATCC
Streptococcus thermophilus RBL 18 FYE 41	n.a.	MH	Lacto-labo, Danisco
Clostridium tyrobutiricum ATCC 25755	n.a.	RCM	ATCC
Lactobacillus acidophilus ATCC 4356	n.a.	MRS	ATCC
Lactococcus lactis ATCC 19257	n.a.	MRS	ATCC

* Lo Verso, L.; Lessard, M.; Talbot, G.; Fernandez, B.; Fliss, I. Isolation and Selection of Potential Probiotic Bacteria from the Pig Gastrointestinal Tract. *Probiotics and Antimicrobial Proteins*, **2017**. https://doi-org.acces.bibl.ulaval.ca/10.1007/s12602-017-9309-3

Table S2. Secondary structure distribution of pediocin PA-1 analogs **1**, **2c**, **3a-c** and **6** in different aqueous TFE solutions and in DMPC and DMPG vesicles based on CD spectroscopy.

Medium	Helix (r)[1]	Helix (d)[2]	Strand (r)	Strand (d)	Turn	Coil	RMSD	NRMSD
**1**								
0% TFE	0.007	0.048	0.081	0.082	0.258	0.525	0.084	0.023
25% TFE	0.064	0.104	0.127	0.094	0.240	0.370	0.104	0.033
50% TFE	0.340	0.170	0.011	0.045	0.143	0.291	0.108	0.017
75% TFE	0.406	0.193	0.000	0.026	0.076	0.245	0.123	0.014
90% TFE	0.646	0.235	0.000	0.001	0.000	0.117	0.068	0.005
DMPC	0.008	0.048	0.060	0.078	0.270	0.537	0.127	0.034
DMPG	0.004	0.013	0.376	0.131	0.180	0.296	0.445	0.341
**2c**								
0% TFE	0.002	0.065	0.000	0.043	0.294	0.595	0.220	0.039
25% TFE	0.070	0.125	0.073	0.065	0.264	0.402	0.096	0.019
50% TFE	0.359	0.200	0.009	0.037	0.117	0.279	0.157	0.020
75% TFE	0.531	0.250	0.000	0.013	0.010	0.196	0.169	0.015
90% TFE	0.693	0.265	0.000	0.000	0.000	0.042	0.104	0.007
DMPC	0.000	0.069	0.034	0.042	0.279	0.575	0.117	0.022
DMPG	0.051	0.001	0.217	0.073	0.218	0.439	0.142	0.032
**3a**								
0% TFE	0.002	0.053	0.220	0.130	0.230	0.366	0.030	0.022
25% TFE	0.002	0.053	0.251	0.127	0.223	0.344	0.031	0.026
50% TFE	0.037	0.074	0.213	0.121	0.227	0.329	0.034	0.025
75% TFE	0.078	0.094	0.191	0.110	0.217	0.31	0.023	0.013
90% TFE	0.125	0.117	0.135	0.097	0.220	0.306	0.029	0.011
**3b**								
0% TFE	0.004	0.058	0.164	0.111	0.240	0.423	0.056	0.022
25% TFE	0.000	0.079	0.175	0.118	0.255	0.372	0.071	0.027
50% TFE	0.114	0.120	0.120	0.091	0.227	0.328	0.056	0.020
75% TFE	0.202	0.162	0.063	0.064	0.202	0.306	0.109	0.028
90% TFE	0.350	0.186	0.007	0.045	0.138	0.275	0.092	0.015
**3c**								
0% TFE	0.002	0.055	0.215	0.126	0.231	0.371	0.055	0.034
25% TFE	0.052	0.069	0.196	0.107	0.229	0.348	0.097	0.052
50% TFE	0.112	0.110	0.142	0.097	0.218	0.321	0.049	0.021
75% TFE	0.191	0.144	0.099	0.080	0.199	0.287	0.078	0.022
90% TFE	0.252	0.159	0.054	0.063	0.176	0.296	0.051	0.012
DMPC	0.001	0.056	0.220	0.131	0.226	0.366	0.056	0.036
DMPG	0.018	0.038	0.285	0.120	0.195	0.345	0.176	0.112
**5**								
0% TFE	0.002	0.061	0.205	0.126	0.230	0.376	0.040	0.021
25% TFE	0.040	0.082	0.223	0.112	0.231	0.313	0.022	0.013
50% TFE	0.113	0.113	0.145	0.095	0.221	0.312	0.036	0.014
75% TFE	0.156	0.139	0.114	0.087	0.209	0.296	0.049	0.017
90% TFE	0.196	0.140	0.096	0.081	0.192	0.295	0.062	0.018
**6**								
0% TFE	0.004	0.044	0.073	0.093	0.257	0.529	0.088	0.024
25% TFE	0.169	0.196	0.056	0.056	0.189	0.333	0.090	0.023
50% TFE	0.174	0.143	0.105	0.090	0.203	0.285	0.129	0.043
75% TFE	0.498	0.200	0.003	0.019	0.044	0.235	0.142	0.015
90% TFE	0.628	0.240	0.000	0.004	0.003	0.125	0.155	0.011
DMPC	0.002	0.046	0.081	0.093	0.269	0.509	0.110	0.031
DMPG	0.027	0.110	0.169	0.114	0.242	0.337	0.125	0.075

[1]**(r)** : Regular, [2]**(d)** : Distorded

Table S3. ^{1}H chemical shifts[a] of pediocin PA-1 M31L (analog **5**) (600 MHz, 313 K, H_2O/TFE-d2 (1:1) containing 0.1% TFA).

	NH	Hα	Hβ	Hγ	Hδ	Hε	Hζ	Hη	$CSD_{H\alpha}$[b]
**Lys1**							-	-	
**Tyr2**	8.408	4.679	2.947	-	7.112	6.795	-	-	0.08
**Tyr3**	8.030	4.525	3.048, 2.777	-	7.067	6.787	-	-	-0.08
**Gly4**	7.003	3.978, 3.765	-	-	-	-	-	-	-0.10
**Asn5**	8.089	4.683	2.834	-	7.320, 6.585	-	-	-	-0.07
**Gly6**	8.281	4.034, 3.894	-	-	-	-	-	-	0.08
**Val7**	7.797	4.272	2.100	0.908	-	-	-	-	0.32
**Thr8**	8.173	4.594	4.179	1.187	-	-	-	-	0.24
**Cys9**	8.387	4.989	3.156, 2.975	-	-	-	-	-	0.34
**Gly10**	8.323	4.040, 3.917	-	-	-	-	-	-	0.07
**Lys11**	8.265	4.165	1.792	1.397	1.690	2.983	-	-	-0.20
**His12**	8.184	4.612	3.360, 3.221	-	7.294	8.500	-	-	-0.02
**Ser13**	7.913	4.544	3.969	-	-	-	-	-	0.04
**Cys14**	8.488	5.029	3.118, 2.965	-	-	-	-	-	0.38
**Ser15**	8.423	4.501	3.857	-	-	-	-	-	0.00
**Val16**	8.085	4.272	1.921	0.907, 0.803	-	-	-	-	0.32
**Asp17**	8.000	4.455	3.233	-	-	-	-	-	0.30
**Trp18**	7.964	4.479	3.366, 3.257	-	7.230	7.521, εNH 9.639	ζ2 7.406, ζ3 7.058	7.168	-0.22
**Gly19**	8.370	3.910, 3.809	-	-	-	-	-	-	-0.16
**Lys20**	7.876	4.211	1.690	1.411	1.584		-	-	-0.15
**Ala21**	7.941	4.111	1.425	-	-	-	-	-	-0.24
**Thr22**	7.798	4.375	4.016	1.250	-	-	-	-	0.03
**Thr23**					-	-	-	-	
**Cys24**				-	-	-	-	-	
**Ile25**						-	-	-	
**Ile26**						-	-	-	
**Asn27**	8.127	4.688	2.839	-	7.352, 6.793	-	-	-	-0.06
**Asn28**	8.135	4.674	2.862, 2.682	-	7.259, 6.558	-	-	-	-0.08
**Gly29**	8.243	3.945	-	-	-	-	-	-	-0.03
**Ala30**	8.116	4.203	1.485	-	-	-	-	-	-0.15
**Leu31**	7.805	4.091	1.775		0.812, 0.571	-	-	-	-0.08
**Ala32**	8.125	4.130	1.429	-	-	-	-	-	-0.22
**Trp33**	7.838	4.412	3.391	-	7.237	7.575, εNH 9.619	ζ2 7.410, ζ3 7.097	7.156	-0.29
**Ala34**	8.110	4.079	1.390	-	-	-	-	-	-0.27
**Thr35**	7.962	4.545	4.176	1.164	-	-	-	-	0.19

**Gly36**			-	-	-	-	-	-		
**Gly37**	8.515	3.936, 3.741	-	-	-	-	-	-	-0.20
**His38**	7.946	4.697	3.319, 3.140	-	7.946	8.510	-	-	0.07
**Gln39**	8.350	4.396	2.376, 2.128	1.979	-	-	-	-	0.03
**Gly40**	8.200	3.930	-	-	-	-	-	-	-0.04
**Asn41**	8.132	4.598	2.758	-	7.333, 6.631	-	-	-	-0.15
**His42**	8.300	4.758	3.289, 3.152	-	7.287	8.527	-	-	0.13
**Lys43**	8.152	4.361	1.853	1.439	1.745	2.992	-	-	0.00
**Cys44**	7.920	4.538	3.295, 3.004	-	-	-	-	-	-0.11

[a] Chemical shifts have been deposited to the wwPDB OneDep System (D_1000225850; PDB ID: 5UKZ).
[b] CSD = Chemical Shift Deviation

Table S4. ^1H-^1H NOEs observed for pediocin PA-1 M31L (analog **5**) (600 MHz, 313 K H_2O/TFE-d2 (1:1) containing 0.1% TFA) that confirm the CS-Rosetta structure.

	AA1	^{1}H1	AA2	^{1}H2	F1 (ppm)	F2(ppm)	Distance (Å)
α-helix	Gly29	NH	Ala32	Hβ	8.243	1.429	2.7
	Leu31	Hδ	Thr35	Hγ	0.571	1.164	4.3
	Gly29	NH	Ala32	NH	8.243	8.125	4.6
	Ala30	NH	Trp33	NH	8.116	7.838	4.7
β-strand	Val7	Hα	Thr8	NH	4.272	8.173	2.1
	Cys9	Hα	Gly10	NH	4.989	8.323	2.2
	Lys11	NH	His12	NH	8.265	8.184	2,4
	Cys14	Hα	Ser15	NH	5.029	8.423	2.4
	Thr8	NH	Ser15	NH	8.173	8.423	3.5
	Gly6	Hα	Asp17	NH	4.034	8.000	3.6
	Gly4	NH	Gly6	NH	7.003	8.281	3.7
	Gly10	NH	Ser15	NH	8.323	8.423	4.4
3-10 helix	Asn41	NH	His42	NH	8.132	8.300	2.2
	Gln39	NH	Gly40	NH	8.350	8.200	2.6
	Gln39	NH	Asn41	NH	8.350	8.132	4.1
Folding (coil)	Ala32	Hα	Gly37	NH	4.130	8.515	2.4
	Val16	$H\gamma_1$	Thr22	Hβ	0.907	4.016	2.5
	Trp18	Hε	Gly19	NH	7.521	8.370	3.4
	Ala32	Hβ	Gly37	NH	1.429	8.515	3.6
	Asn28	Hδ	Gln39	Hβ	7.259	2.376	3.6
	Val16	$H\gamma_2$	Thr22	Hβ	0.803	4.016	4.0
